# Lipo-chitooligosaccharides as regulatory signals of fungal growth and development

**DOI:** 10.1038/s41467-020-17615-5

**Published:** 2020-08-04

**Authors:** Tomás Allen Rush, Virginie Puech-Pagès, Adeline Bascaules, Patricia Jargeat, Fabienne Maillet, Alexandra Haouy, Arthur QuyManh Maës, Cristobal Carrera Carriel, Devanshi Khokhani, Michelle Keller-Pearson, Joanna Tannous, Kevin R. Cope, Kevin Garcia, Junko Maeda, Chad Johnson, Bailey Kleven, Quanita J. Choudhury, Jessy Labbé, Candice Swift, Michelle A. O’Malley, Jin Woo Bok, Sylvain Cottaz, Sébastien Fort, Verena Poinsot, Michael R. Sussman, Corinne Lefort, Jeniel Nett, Nancy P. Keller, Guillaume Bécard, Jean-Michel Ané

**Affiliations:** 1 Department of Bacteriology, University of Wisconsin–Madison, Madison, WI 53706 USA; 2 Department of Agronomy, University of Wisconsin–Madison, Madison, WI 53706 USA; 3 Laboratoire de Recherche en Sciences Végétales, Université de Toulouse, CNRS, UPS, Castanet-Tolosan, France; 4 Laboratoire Évolution et Diversité Biologique, Université de Toulouse, CNRS, UPS, IRD, Toulouse, France; 5 Laboratoire des Interactions Plantes-Microorganismes, Université de Toulouse, INRAE, CNRS, Castanet-Tolosan, France; 6 Department of Medical Microbiology and Immunology, University of Wisconsin-Madison, Madison, WI 53706 USA; 7 Department of Medicine, University of Wisconsin–Madison, Madison, WI 53706 USA; 8 Biosciences Division, Oak Ridge National Laboratory, Oak Ridge, TN 37831 USA; 9 Department of Microbiology, University of Tennessee, Knoxville, TN 37996 USA; 10 Department of Chemical Engineering, University of California, Santa Barbara, CA 93106 USA; 11 Univ. Grenoble Alpes, CNRS, CERMAV, 38000 Grenoble, France; 12 Laboratoire des Interactions Moléculaires et Réactivités Chimiques et Photochimiques, Université de Toulouse, CNRS, UPS, Toulouse, France; 13 Department of Biochemistry, University of Wisconsin–Madison, Madison, WI 53706 USA; 14 Present Address: Bioscience Division, Oak Ridge National Laboratory, Oak Ridge, TN 37831 USA; 15 Present Address: South Dakota State University, Brookings, SD 57007 USA; 16 Present Address: North Carolina State University, Raleigh, NC 27695 USA; 17 Present Address: University of Georgia, Athens, GA 30602 USA

**Keywords:** Microbial ecology, Cellular microbiology, Fungal biology, Fungal ecology

## Abstract

Lipo-chitooligosaccharides (LCOs) are signaling molecules produced by rhizobial bacteria that trigger the nodulation process in legumes, and by some fungi that also establish symbiotic relationships with plants, notably the arbuscular and ecto mycorrhizal fungi. Here, we show that many other fungi also produce LCOs. We tested 59 species representing most fungal phyla, and found that 53 species produce LCOs that can be detected by functional assays and/or by mass spectroscopy. LCO treatment affects spore germination, branching of hyphae, pseudohyphal growth, and transcription in non-symbiotic fungi from the Ascomycete and Basidiomycete phyla. Our findings suggest that LCO production is common among fungi, and LCOs may function as signals regulating fungal growth and development.

## Abstract (web summary)

Lipo-chitooligosaccharides (LCOs) are signaling molecules produced by certain bacteria and fungi that establish symbiotic relationships with plants. Here, the authors show that LCOs are produced also by many other, non-symbiotic fungi, and regulate fungal growth and development.

## Introduction

A conceptual leap in our understanding of the mechanism of plant-microbe symbiosis came when, almost 30 years ago, nitrogen-fixing rhizobial bacteria were found to produce nodulation (Nod) factors that are required to induce the formation of nodules on legume roots^1^. These Nod factors are lipo-chitooligosaccharides (LCOs), which consist of a polymer of three to five *N*-acetyl glucosamine (GlcNAc) residues (the chitin backbone) with β-(1,4) linkages modified with a long-chain fatty acyl group and various other functional groups^2^. Most rhizobia rely on LCOs to associate with their legume hosts and legumes can perceive LCOs down to 10^−14^ M concentrations^3^. Substitutions on the chitinous backbone are largely responsible for the often high level of host specificity observed in the rhizobia-legume symbiosis^2^.

Nearly 20 years later, arbuscular mycorrhizal (AM) fungi (sub-phylum Glomeromycotina), which are another group of microorganisms that live symbiotically with plant roots, were also found to produce LCOs (Myc-LCOs)^4^. Additional studies have shown that these two dissimilar symbioses share a highly conserved Common Symbiosis Signaling Pathway (CSSP), which is activated in plants by Nod- or Myc-LCOs to allow root colonization (either Nod or mycorrhization, respectively^5,6^). In both symbioses, LCOs are perceived at the plasma membrane by receptor-like kinases with extracellular LysM domains^7,8^. The perception of short chitooligosaccharides (COs) is also required to initiate the AM association and is mediated by the same family of LysM-containing receptors^7^.

We recently reported that a representative from a third group of symbiotic microorganisms, the ectomycorrhizal (EM) fungus *Laccaria bicolor* (phylum Basidiomycetes), also synthesizes LCOs^9^. *L. bicolor* colonizes the roots of *Populus*, a host plant that contains the genetic components of the CSSP and can also be colonized by AM fungi^9^; however, another EM fungus that was suspected to produce LCOs, *Hebeloma cylindrosporum*, colonizes mostly pine, which does not contain the components for the CSSP^10^. This latter finding suggests that LCOs may have functional roles beyond symbiotic signaling.

In this study, we explored the possibility that the production of LCOs is a more common trait among fungi than was previously anticipated. We demonstrate that LCOs are not produced solely by symbiotic microorganisms but also by widely divergent members of the Kingdom Fungi. Moreover, we show that LCOs have regulatory functions in fungal development.

## Results

### LCOs are produced by a wide range of fungi in the Kingdom Fungi

**Fig. 1:**
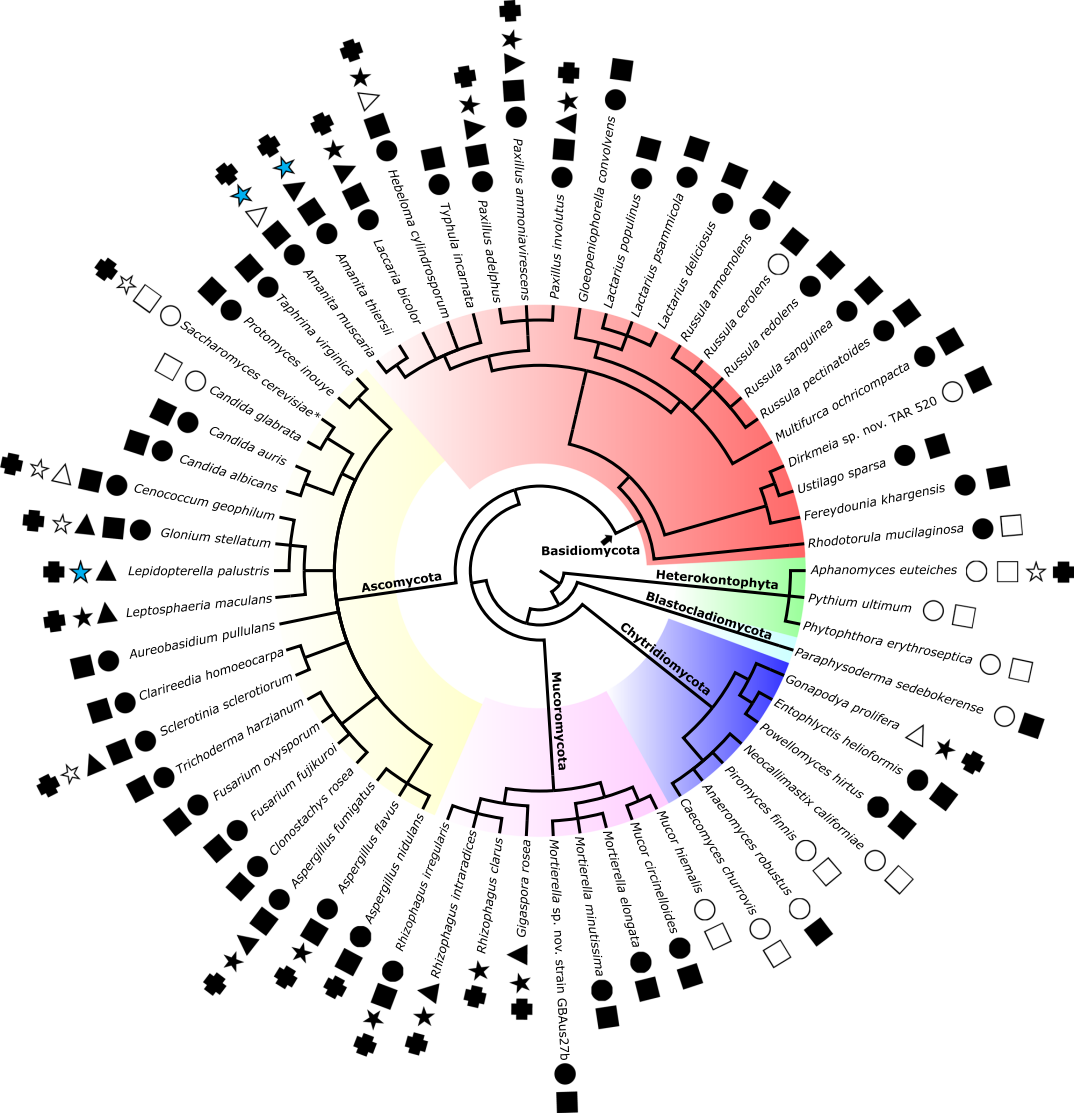
Production of lipo-chitooligosaccharides and chitooligosaccharides by fungi. Fifty-nine fungi representing five of the eight phyla (indicated by colors) and three species of oomycetes (Heterokontophyta, green) were tested for the presence of lipo-chitooligosaccharide (LCO)s and chitooligosaccharide (CO)s in their culture supernatants. Black circle, detection of sulfated LCOs by the root hair branching assay with *M. truncatula*. Black square, detection of non-sulfated LCOs by the root hair branching assay with *V. sativa*. Black triangle, detection of sulfated LCOs in butanol extracts by *MtENOD11* expression assay. Black star, detection of LCOs in butanol extracts by LC-MS/MS with high confidence (non-targeted mass spectrometry (MS) analysis or two to three MRM transitions per molecule). Blue star, detection of LCOs with lower confidence (MS signal at the expected retention time but with only one MRM transition). Black cross, detection of COs by HPLC/MS from water extracts. Clear symbols indicate no detection. Black asterisk indicates two or more strains were examined.

We tested 59 species of fungi belonging to most phyla within the kingdom for the presence of LCOs exuded into their culture media (Fig. 1 and Supplementary Data 1, 2, and 3). These exudates were assayed for LCO activity using the highly sensitive root hair deformation response triggered by LCOs in barrel medic (*Medicago truncatula*) and common vetch (*Vicia sativa*)^1,2^. As some of these root hair deformations, such as waving or bulb formation, are not specific responses to LCOs, our assay was scored strictly on root hair branching. In control experiments, as expected from their known Nod factor specificities, *M. truncatula* responded only to sulfated (s) LCOs, whereas *V. sativa* responded only to non-sulfated (ns) LCOs^2^ (Supplementary Fig. 1). To test the specificity of the root hair branching response to LCOs, we examined the effect of short COs, polymers of four to five GlcNAc residues (CO4 and CO5), which are precursors of LCOs and have been shown to also activate the CSSP^11,12^. We also tested long CO chains (CO8), which are not LCO precursors, but oligomers that activate symbiotic and defense-related responses^7,13^. Root hair branching was not triggered by the application of short (CO4 and CO5) or long (CO8) chain COs, fatty acids (palmitic or oleic), or by the fresh culture media in which the fungi grew (Supplementary Fig. 1 and Supplementary Data 4). Also, the absence in the fungal samples of bacteria that might produce LCOs was verified with specific PCR amplification (using fungus-specific and bacterium-specific primers) and light microscopy observation (Supplementary Fig. 2). When the exudates from 53 fungi were applied to the roots of *M. truncatula* or *V. sativa*, 47 of them triggered root hair branching in one or two legumes (Fig. 1, Supplementary Figs. 3–7, and Supplementary Data 4). We confirmed the presence of sLCOs in some butanol extracts of exudates by assaying for expression of the *MtENOD11* gene in *M. truncatula*^4^ (Fig. 1 and Supplementary Fig. 8). Interestingly, the exudates of the yeasts *Saccharomyces cerevisiae* and *Candida glabrata* did not induce root hair branching (Fig. 1). Compared with *Candida albicans* or *Candida auris* in which we detected LCO activity (Fig. 1), *C. glabrata* is more closely related to *S. cerevisiae* and reported to form pseudohyphae only under stress conditions or when genetically altered^14,15^.

### The structure of fungal LCOs

**Fig. 2:**
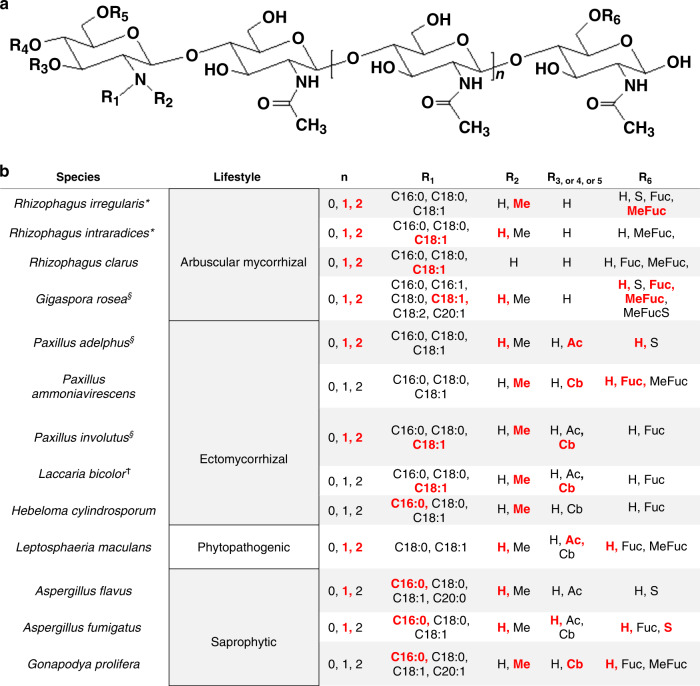
Structures of LCOs found in fungi. **a** The generic structure of LCOs. **b** LCO structures determined by LC-MS/MS analysis of the butanol phase extract of culture media from fungi with various lifestyles. Red indicates the most abundant LCO structures. (§) indicates when untargeted mass spectrometry (MS) analysis was used. The other structures were detected in targeted MS mode (MRM) (see “Methods” for details of various possible MRM transitions). (*) indicates when more than one strain was analyzed. (n) is the number of residues of chitin oligomers, (R1) is the type of fatty acid, identified as saturated or unsaturated fatty acids. (R2–R6) are chemical substitutions: hydrogen (H), acetyl (Ac), carbamoyl (Cb), fucosyl (Fuc), fucosyl sulfate (FucS), methylfucosyl (MeFuc) and sulfate (S). (ϯ) indicates data published previously^9^.

To confirm our findings with the root hair branching assay for LCOs and to determine the structure of LCOs produced by the various fungi, we used mass spectrometry (MS). The culture media were fractionated by butanol:water phase separation. The water phases were analyzed directly for COs and the butanol phases, in which LCOs were expected to fractionate, were either analyzed directly or were further purified by chromatography to minimize matrix effects on the MS analyses. Taking advantage of an in-house database (Supplementary Data 5) listing all possible mass-to-charge ratios (precursors/products ions) calculated from known Nod factors, and by using the very sensitive Multiple Reaction Monitoring (MRM) MS approach, we found mass signals of LCOs in 16 of the 20 fungal exudates we analyzed (Fig. 1). The LCOs contained three to five GlcNAc residues bearing various fatty acyl chains and additional sulfate, methyl, carbamoyl, fucosyl, and methylfucosyl substitutions (Fig. 2). Exudates from three fungal species that were LCO positive—*Gigaspora rosea*, *Paxillus adelphus*, and *Paxillus involutus*—were concentrated enough for untargeted LCO detection by using enhanced MS-enhanced product ion (EMS–EPI), allowing a more exhaustive analysis of the different structures present. Examples of chromatograms and spectra of major LCO structures found in these three fungal exudates are given in Supplementary Figs. 9–11. LCOs were not detected in exudates from *Cenococcum geophilum*, *Glonium stellatum*, *Saccharomyces cerevisiae,* and *Sclerotinia sclerotiorum* (Fig. 1). Notably, the chemical structures of all detected LCOs were quite similar across the Kingdom Fungi, with the fucosyl and methylfucosyl functional groups as the most commonly found, even in species of AM fungi. In the water phases of the 20 analyzed fungal exudates, we found mass signals corresponding to COs containing three to five GlcNAc residues.

Given that the oomycete plant pathogen *Aphanomyces euteiches* produces COs^16^, we also analyzed exudates of this organism for the presence of chitinous molecules by MS and exudates of other Heterokontophyta/oomycete representatives *Pythium ultimum* and *Phytophthora erythroseptica* by using root hair branching assay. We confirmed the presence of short COs by MS but detected no LCOs either by MS or by the functional assays, suggesting that the ability to produce LCOs may be restricted to rhizobial bacteria and to the Kingdom Fungi (Fig. 1).

### LCOs have regulatory functions in fungal development

**Fig. 3:**
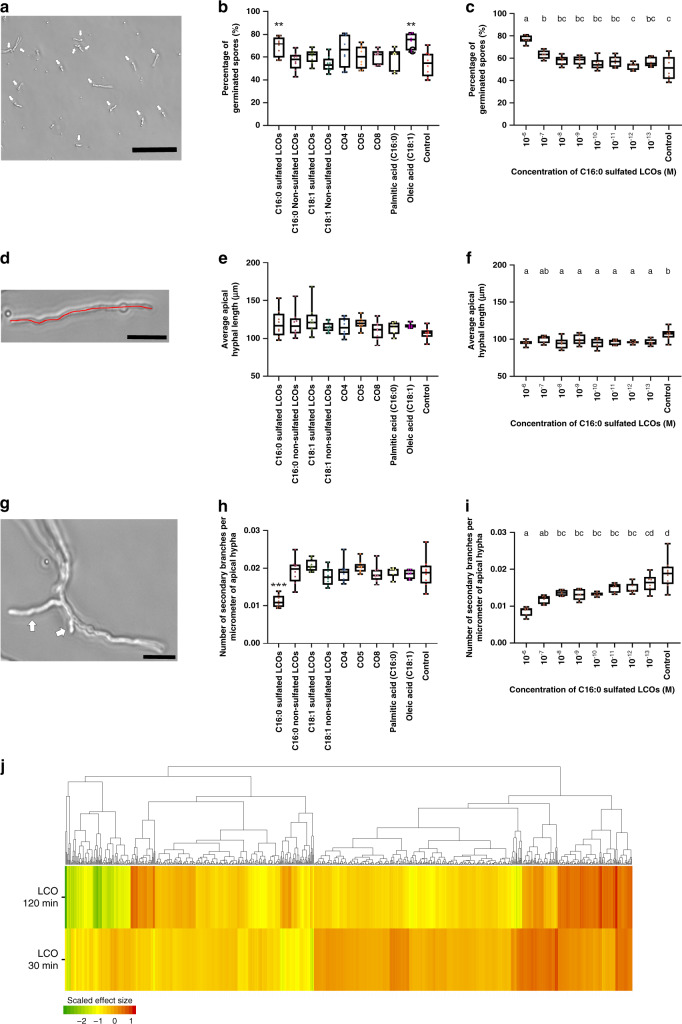
Effects of LCOs, COs, and fatty acids on *A. fumigatus*. **a** Germinated spores are indicated by white arrows. The scale bar is 100 µm. **b** Percentage of germinated spores 10 h after treatment with various molecules at 10^−8^ M. (**) indicates a significant difference between C16:0 sulfated LCO or oleic acid treatments and the control according to Dunnett’s multiple comparison procedure, the *p*-value is 7.21 × 10^−4^. Each treatment was analyzed in eight independent wells, except the palmitic and oleic acid treatments that were analyzed in six of them. **c** Effect of a range of C16:0 sulfated LCO concentrations on the percentage of germinated spores; one-way ANOVA *p*-value of 6.32 × 10^−8^. Each LCO concentration was analyzed in six wells, except for the control treatments that were analyzed in eight of them. **d** An example of an apical hypha germinated from both sides of a spore whose length measured is indicated in red. Scale bar is 25 µm. **e** Length of apical hyphae after 12 h treatment with various molecules at 10^−8^ M. There were no significant differences between treatments. Each treatment was analyzed in eight independent wells, except palmitic and oleic acid treatments that were analyzed in six of them. **f** Effect of a range of C16:0 sulfated LCO concentrations on the apical hyphae length; one-way ANOVA *p*-value is 3.91 × 10^−3^. Each LCO concentration was analyzed in six independent wells, except the control which was analyzed in eight of them. **g** Germination of a control spore showing two secondary branches (arrows) on a germinating apical hypha. Scale bar is 25 µm. **h** The ratio of secondary branches per micrometer of apical hypha after 12 h treatment with various molecules at 10^−8^ M. (***) indicates a significant difference between C16:0 sulfated LCO treatments and the control according to Dunnett’s multiple comparison procedure, *p*-value is 5.89 × 10^−9^. Each treatment was analyzed in eight independent wells, except palmitic and oleic acid treatments, which were analyzed in six of them. The ratio was determined by calculating the number of secondary branches for that specific apical hyphal branch length. **i** Effect of a range of C16:0 sulfated LCO concentrations on the ratio of secondary branches; one-way ANOVA *p*-value is 1.54 × 10^−9^. Each LCO concentration was analyzed in six independent wells, except the control which was analyzed in eight of them. The ratio was determined by calculating the number of secondary branches for that specific apical hyphal branch length. In box plots (**c**, **f**, **i**), different letters indicate significant differences and similar letters indicate no difference according to Tukey’s single-step multiple comparison procedure. In the box plots (**b**, **c**, **e**, **f**, **h**, **i**), the bars represent the minimum value, the first quartile, the median, the third quartile, and the maximum value such that 25% of the data are in each section. **j** Heatmap showing the scaled effect size of differentially expressed genes 30 and 120 min after C16:0 sulfated LCO treatment at a concentration of 10^−8^ M compared with the control solution. Source data are provided as a Source Data file.

We found LCOs in fungi with various lifestyles and under different growth conditions (Figs. 1 and 2, and Supplementary Data 1, 2, and 3), including in non-symbiotic saprotrophic fungi that live and feed on dead organic matter, and in pathogenic fungi that grow on animals or plants, suggesting that their roles in fungal biology are not limited to symbioses with plants. To explore this hypothesis, we applied synthetic sLCOs or nsLCOs with various fatty acyl chains (C16:0—palmitic acid or C18:1—oleic acid), short (CO4 and CO5) and long (CO8) COs, and the C16:0 and C18:1 fatty acids to the saprotrophic and opportunistic human pathogen *Aspergillus fumigatus*, which exhibits two easily scored developmental processes, germination, and hyphal branching. C16:0 sLCO (10^−8^ M) and oleic acid (10^−8^ M) increased spore germination by 28% and 36%, respectively, when compared with the control (Fig. 3a, b). The response to LCOs was dose dependent (Fig. 3c). Although the length of the primary apical hyphae was similar across the different treatments (Fig. 3d, e), not exceeding 11% difference among them (Fig. 3f), the branching of lateral hyphae, treated with the C16:0 sLCO, decreased by up to 41% (Fig. 3g, h) and in a dose-dependent manner (Fig. 3i). The activity of C16:0 sLCO was detected at as little as 10^−12^ M. As the saccharidic (COs) and lipidic (oleic and palmitic acids) moieties of LCOs were not acting alone, we conclude that the observed biological activity of C16:0 sLCO is linked to its lipo-chitooligosaccharidic nature. As germination and branching are programmed developmental processes in fungi, we investigated the effect of these molecules on transcription activity in *A. fumigatus*. We found 91 differentially expressed genes (DEGs) between control and LCO-treated cultures after only 30 min of treatment and 152 DEGs after 120 min (Fig. 3j, Supplementary Fig. 12, and Supplementary Data 6). Gene Ontology (GO) and KEGG (Kyoto Encyclopedia of Genes and Genomes) pathway analyses revealed that several DEGs encoding proteins associated with the cell membrane activities and cell wall processes were regulated at 30 min after treatment with LCOs. A much more diverse set of functions was represented at 120 min with a large number of proteins associated with responses to chemical stimuli and enzymes related to the tricarboxylic acid cycle (Supplementary Data 7 and 8). The vast majority of regulated genes in response to LCOs increased in expression and there was a significant overlap in these upregulated genes at 30 and 120 min, suggesting that some of these genes could be good reporters for the response to LCOs in *A. fumigatus* (Supplementary Fig. 13).

**Fig. 4:**
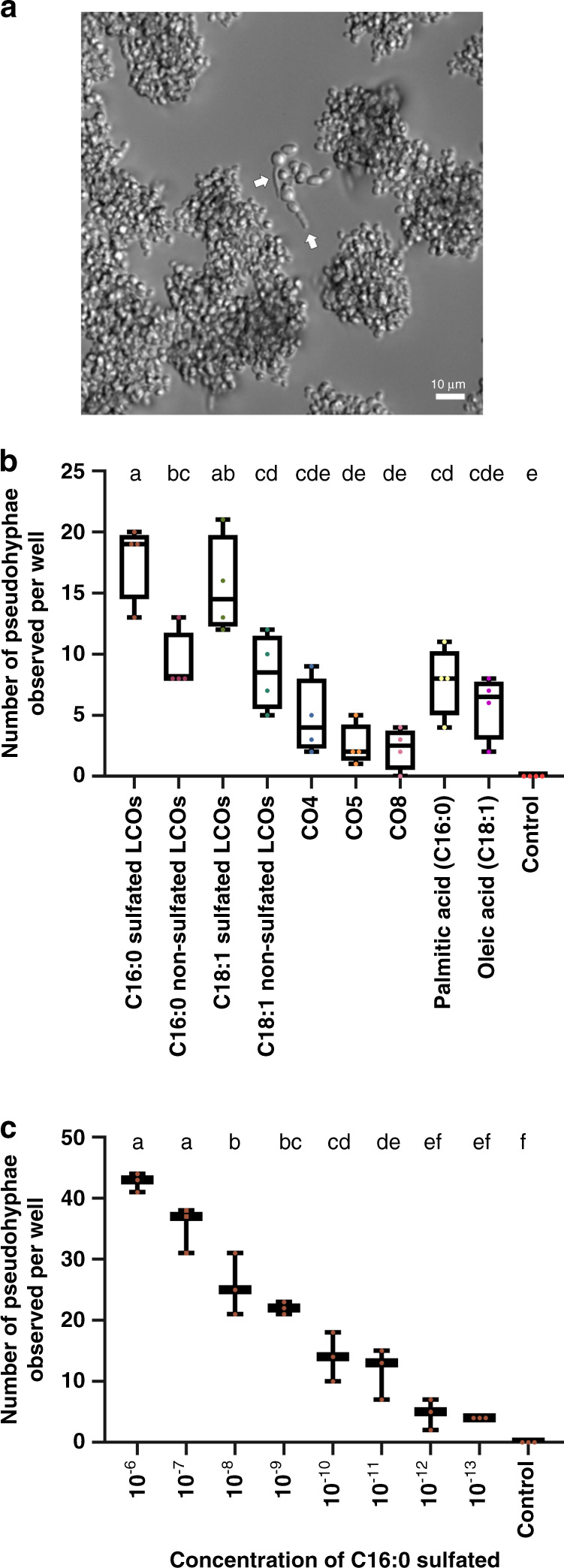
Effect of LCOs, COs, and fatty acids on *C. glabrata*. **a** White arrows show pseudohyphae of *C. glabrata* (see also Supplementary Movies 1, 2, and 3). Scale bar is 10 µm. **b** The number of pseudohyphae observed per well after treatment for 12 h with various LCOs, COs, and fatty acids at 10^−8^ M. Different letters indicate significant differences and similar letters indicate no difference according to Tukey’s single-step multiple comparison procedure; one-way ANOVA *p*-value is 9.90 × 10^−10^. Each treatment was analyzed in four independent wells. **c** Effect of a range of C16:0 sulfated LCO concentrations on pseudohyphae formation; one-way ANOVA *p*-value is 4.67 × 10^−12^. Different letters indicate significant differences and similar letters indicate no difference according to Tukey’s single-step multiple comparison procedure. Each LCO concentration was analyzed in three independent wells. In the box plots (**b**, **c**), the bars represent the minimum value, the first quartile, the median, the third quartile, and the maximum value such that 25% of the data are in each section. Source data are provided as a Source Data file.

We also tested the effect of 10^−8^ M LCOs, COs, oleic, and palmitic acids on the growth of *C. glabrata*, a yeast in which we did not detect the synthesis of LCOs. All the molecules tested stimulated the formation of pseudohyphae (Fig. 4a, b and Supplementary Movies 1, 2, and 3), but the greatest activity was observed with the C16:0 sLCO after 12 h of exposure. This effect was dose dependent and detected at as little as 10^−13^ M (Fig. 4c and Supplementary Movie 1).

To evaluate whether fungi outside the phylum Ascomycota respond to LCOs, we tested a Basidiomycete that is a common allergen in humans, the yeast *Rhodotorula mucilaginosa*. Treatment with 10^−8^ M C16:0 sLCOs led to a 25% increase in the proliferation of *R. mucilaginosa* cells (Supplementary Fig. 14).

## Discussion

The findings reported here impose a paradigm shift in our understanding of the biology of LCOs. Until now, these molecules were considered as exclusively produced by plant microbial symbionts, rhizobia, AM, and EM fungi. We now show that nearly all fungi—not only those that interact with plants—produce LCOs whose structures are very similar to those of Nod factors. As LCOs have been shown to suppress innate immunity in plants^7,17,18^, a function that may have predated the mycorrhizal symbiosis^19^, this discovery raises questions on how plants can distinguish symbiotic microbes from pathogenic fungi. Although it is indisputable that LCOs are symbiotic signals in the sense that they activate the CSSP pathway in plants, it remains to demonstrate that they are also used by rhizobia and mycorrhizal fungi to be distinguished from non-symbiotic microorganisms. Interestingly, the production of LCOs in rhizobia and of precursors of LCOs (short COs) in AM fungi are strongly stimulated by specific root signals^2,12^. This could ensure the production of the right LCO structures, in the right place, at the right time, and with adequate concentrations. If the existence of such a molecular dialogue also exists in non-symbiotic interactions, then additional symbiotic signals, other than LCOs, must provide specificity. AM fungi may produce more distinctive symbiotic signals, yet to be discovered, whereas pathogenic fungi are known to produce additional effectors recognized by the immune system of plants. In the former case, the ligand of the plant Dwarf14-Like (D14L) receptor that activates the D14L-dependent signaling mechanisms and the ensued removal of the negative regulator of mycorrhization SMAX1, could be a good candidate^20,21^. In the case of pathogenic fungi, the role that LCOs and COs could play in their virulence should be investigated. Indeed, these molecules, particularly when they are combined, can act synergistically to enhance symbiosis and suppress immunity^7^, i.e. can have an inverse role to that of the traditional pathogen-associated molecular pattern molecules also produced by the same organisms.

Given that we have detected LCOs in widely divergent lineages of fungi that diverged before the first land plants, we speculate that LCO production is an ancestral trait of fungi and that plants have acquired the ability to recognize these molecules first to detect the close presence of a fungus, then as symbiotic signals^19^. This hypothesis is in line with the recent reports that the same LysM-containing plant receptors are involved in both immunity and symbiosis signaling^7,16,22^. Our findings could also explain the surprising data showing that LCOs are active on mammalian cells^23^.

The effect of LCOs on *A. fumigatus* and *C. glabrata* development was observed at concentrations down to 10^−12^ M and 10^−13^ M, respectively. It seems unlikely that LCOs will have a nutritional effect at such low concentrations but we cannot exclude that building blocks or degradation products of LCOs (chitin oligomers and fatty acids) may also play a regulatory role. Indeed, we observed some effect of COs and fatty acids on fungal germination of *A. fumigatus* and on the formation of pseudohyphae in *C. glabrata*, but these effects were generally more limited than those of C16:0 sLCO.

Rather than nutrients, our work strongly supports LCOs as representing fungal autocrine and paracrine signals. We showed that *A. fumigatus* and *C. glabrata* responded to LCOs in a dose-dependent and structure-dependent manner. Furthermore, we find that cell membrane and perception genes are significantly upregulated within 30 min of *A. fumigatus* exposure to LCOs, processes associated with response to signal molecules. If the production of these molecules in the environment is proportional to fungal cell density, they could have a function similar to quorum-sensing molecules in bacteria or yeasts, but this concept is difficult to define with filamentous fungi^24–26^. It seems likely that LCO production will vary with life stages. Unfortunately, the detection techniques that we used (root hair deformation, gene expression, and MS analyses) are specific and sensitive but not quantitative enough to further investigate this quorum-sensing hypothesis.

Before this study, pseudohyphae in *C. glabrata* had only been observed under harsh conditions^27,28^. Interestingly, infections with *C. glabrata* have often been reported in the presence of *C. albicans*, a fungus that produces LCOs (Fig. 1^14,29^). It would be interesting to examine if the production of pseudohyphae and the pathogenicity of *C. glabrata* may be regulated by the perception of LCOs produced by other fungi.

We cannot exclude, at this point, that LCOs may have some unspecific structural effect on the fungal cell wall but the timeframe of the transcriptional response to these molecules (<30 min) as well as the low concentration (10^−8^ M) sufficient to elicit their biological activities suggest that LCOs may be perceived by specific receptors. Given that LCOs are perceived by LysM receptor-like kinases in plants, it is tempting to speculate that LysM-containing proteins may also be involved in LCO perception in fungi^8^. As shown in Supplementary Table 1, fungi possess a significant number of LysM-containing proteins whose functions are largely unknown. It will also be interesting to decipher the signaling pathways in fungi controlling the transduction of the LCO signals. Investigating these questions will require new model organisms, such as *A. fumigatus* and *C. glabrata*, amenable to reverse genetics that may reveal new, perhaps essential, roles for LCOs in the biology of fungi.

Many questions remain such as how and where LCOs are synthesized in fungi. It will be interesting to determine if they are anabolically produced, like in rhizobia, or produced from the degradation and modification of longer chitin molecules. As a minimum, chitin synthases, chitin deacetylases, and *N*-acyltransferases are the enzymes required to produce the backbone of LCOs in fungi. An estimate for the number of genes encoding such proteins in Ascomycetes, Basidiomycetes, and Zygomycetes is provided in Supplementary Table 1. Based on these numbers, chitin deacetylase and perhaps chitin synthases would be the most obvious targets to search for mutants unable to produce LCOs. Such an approach would obviously be essential to uncover the fundamental and probably conserved roles played by LCOs in the development of fungi and in their interaction with the biotic environment.

## Methods

### Analysis of COs and LCOs from fungal exudates

The list and sources of the 59 species of fungi and three species of oomycetes (Heterokontophyta) used in the study are presented in Supplementary Data 1–3. The fungal species examined are representatives of each sub-phyla within five phyla (out of eight phyla) of the Kingdom Fungi^30^. The absence of contaminants in the fungal and oomycete strains was systematically checked by PCR by using the specific primers ITS1F/ITS4 and fD1/rP2^31,32^ (Supplementary Fig. 2). The inoculum type (cells, mycelium, spores, or zoospores), culture media and culture times used for each strain are indicated in Supplementary Data 1–3.

Fungi and oomycetes producing mycelia were pre-cultivated in Petri dishes on the solid media gelled with agar as indicated in Supplementary Data 2^33,34^. Once the mycelium had covered the dish, plugs of the mycelium were transferred to Sylon-coated culture flasks^35^ or to 6.7 × 11.4 cm flat-bottom PYREX® flasks (Corning, Inc. Corning, NY), or for the Russulales to 25 × 95 mm flat-bottom culture tubes (PhytoTech), respectively, filled with 50 ml or 12 ml of the appropriate liquid medium to produce and collect exudates (Supplementary Data 2). In addition, using a separate experimental method, *C. geophilum*, *G. stellatum, Lepidopterella palustris, Leptosphaeria maculans*, *S. sclerotiorum*, and the species of *Amanita*, *Hebeloma*, and *Paxillus* were pre-cultivated as above but they were inoculated on a cellophane membrane laid on the solid medium. This membrane was used to transfer agar-free mycelium to Petri dishes filled with deionized sterile water or with liquid culture medium in order to produce and collect exudates (Supplementary Data 2).

For the anaerobic *Neocallimastigomycetes*, *Neocallimastix californiae*, *Piromyces finnis*, *Anaeromyces robustus*, and *Caecomyces churrovis*, 1 ml of fungal zoospores was used to inoculate 20 ml of modified minimal Medium C in a 60 ml borosilicate serum bottles containing 0.2 g switchgrass while sparging with CO_2_^36,37^. Fungal cultures were incubated anaerobically 6 days before collecting the exudates.

For fungi (except AM fungi) producing cells, spores, or zoospores, 10^6^ of these propagules were produced and collected according to published methods^33,37–48^. Propagules were inoculated directly in five independent Sylon-coated flasks with 50 ml liquid medium per species. AM cultures were propagated by in vitro mycorrhizal root organ cultures in solid M medium containing Phytagel (Sigma-Aldrich) and collected after solubilization of Phytagel^39,49^. Exudates from the AM fungal strains were collected from 10,000 spores germinating in 10 ml liquid medium for 10 days.

The various liquid media (broth or water), enriched with exudates, were filtered under sterile conditions through a 0.22 µm Millipore Express® PES membrane (MilliporeSigma, Darmstadt, Germany) prior to being analyzed in the bioassays.

One hundred to 400 ml of culture filtrates, depending on the fungal cultures, were extracted twice with butanol (1 : 1 v/v). The pooled butanol phases were washed with distilled water and evaporated under vacuum. The dry extract was re-dissolved in 4 ml water : acetonitrile (ACN) (1 : 1 v/v) and dried under nitrogen. This crude extract was resuspended in 1 ml of 20% ACN in water and separated on Hypersep C18 (500 mg, 3 ml, Thermo Fisher Scientific) by sequential elution with 3 ml each of 20%, 50%, and 100% ACN in water, respectively. The eluted samples were then dried under nitrogen. Occasionally, for further purification, the 50% eluate was resuspended in 75% ACN in water and separated on Chromabond HILIC (500 mg, 3 ml) by sequential elution with 3 ml each of 100%, 80 and 75% ACN in water. The eluates were then dried under nitrogen.

The presence of LCOs in filtered crude exudates (1× or 10×) or in the butanol fractions of media were assayed by root hair branching in *V. sativa*, which is induced by nsLCOs^50^, by root hair branching in *M. truncatula* accession Jemalong A17, which is induced by sLCOs, and by expression of *MtENOD11* using the *pENOD11:GUS* transcriptional fusion in *M. truncatula*, which is also induced by (s)LCOs^4,51^.

The root hair branching assays in *V. sativa* and *M. truncatula* used the method of Cope et al.^9^. Eight young seedlings (3–7 days old) were treated with the fungal exudates, with the same concentration of solvent (negative controls), or with Nod factors purified from *Rhizobium leguminosarum* biovar *viciae* or *Sinorhizobium meliloti* supernatant at a concentration of 10^−8^ M (positive controls). One milliliter of fungal crude exudates or 40 µl of butanol fractions were applied on each seedling primary root.

The *MtENOD11* gene expression assay was performed as in Maillet et al.^4^. Two kinds of samples were tested: butanol extracts diluted 100 times in water and HILIC column fractions diluted 10 times. Forty microliters of these solutions were applied to the primary root of each seedling for 16 hours. Seven to ten seedlings were tested by sample and compared to mock treatment (0.005% EtOH in water or 5% ACN in water). Plants were stained for 6 h. An arbitrary scale was used to quantify GUS (beta-glucuronidase)-staining (Supplementary Fig. 8).

Standard LCO compounds (non-sulfated C16:0 LCO IV, sulfated C16:0 LCO IV, non-sulfated C18:1 LCO IV, sulfated C18:1 LCO IV) were synthesized at CERMAV (Grenoble, France) and were used at 10^−5^ M in ACN/water (1/1, v/v) to determine retention times and to optimize HPLC/QTRAP tandem MS detection by MRM^4,12^. The UltiMate 3000 HPLC system (Dionex Corporation) was equipped with an Acquity C18 reversed-phase column (2.1 × 100 mm, 1.7 µm, Waters Corporation). Samples of 10 µl were injected. The elution was done at a constant flow rate of 450 µl min^−1^ using solvent A, water:acetic acid (1000 : 1, v-v) and solvent B, ACN, as follows: 30% B for 1 min, followed by a 30–100 % B during 8 min, followed by isocratic elution with 100% B for 2 min. A QTRAP 4500 mass spectrometer (Applied Biosystems, Foster City, USA) equipped with an electrospray ionization source in the positive ion mode was used to analyze samples in the MRM mode or in the EMS–EPI mode (see below). For the MRM mode analyses, from the known substitutions and chitin lengths already described for Nod factor structures, we created a database of all possible combinations of structures, including new ones never described before, with their corresponding precursor proton adduct ion [M + H]^+^ and product B ions: in total, 76,386 precursor ions, 2,598,159 theoretical combined structures, and 358,473 MRM transitions (Supplementary Data 5). Given that the number of MRM transitions to be selected for each analysis must be reasonably low to ensure proper sensitivity, we have selected the most commonly described Nod-LCOs (corresponding to 990 MRM transitions). This highly sensitive, targeted, analytical approach was suitable for samples containing low concentrations of molecules. For samples with higher concentrations of molecules, full scan EMS–EPI analyses were performed. During EMS analysis, major precursor ions are selected automatically, and, after the collision, EPI analysis accumulates their product ions in the trapping module. From this data set, we selected only the precursor ions containing 3 to 6 GlcNAc. This more comprehensive mode could only be used with *P. adelphus*, *P. involutus*, and *G*. *rosea* LCO-rich samples.

Short COs were separated and analyzed using the same LC-MS system, equipped with an hypercarb column (5 μm, 2 × 100 mm; Hypercarb, Thermo). Samples of 10 µl were injected. The elution was done at a constant flow rate of 400 µl min^−1^ using solvent A, water : acetic acid (1000 : 1, v-v) and solvent B, ACN, as follows: 100% A for 1 min, then 100–50% A in 30 min then 50–0% A in 3 min. COs were identified in the MRM mode by monitoring the transitions from precursor proton adduct ion [M + H]^+^ *m/z* 628 (CO3), 831 (CO4), 1034 (CO5), or 1237 (CO6) generating after collision-induced dissociation (CID) the common product B ion *m/z* 204, comparatively to standard solutions (10^−7^ M in water). The capillary voltage was fixed at 4500 V and the source temperature at 400 °C. Fragmentation was performed by CID with nitrogen at a collision energy of 22–54 V; declustering potential was 90–130 V, optimized for each synthetic molecule available. Data processing was performed using Analyst 1.6.1 software (AB Sciex).

### Experiments with *A. fumigatus*

*A. fumigatus* strain Af293 was grown in standard 90 mm Petri dishes on solid glucose minimal medium (GMM) and placed in the dark at 37 °C for 48 h^52^. Ten milliliters of 80% Tween 20 (Acros Organics, New Jersey) in sterile MiliQ water were added to the dishes and agitated with a sterile L-shaped cell spreader (Thermo Fisher Scientific, Waltham, MA) to collect spores. The spore suspension was sterilely transferred to a 50 ml polypropylene sterile Falcon® Centrifuge Tubes (Corning, Corning, NY). The spore suspension was homogenized by vortexing at maximum speed, and a 1 : 10 dilution was prepared with sterile MiliQ water, which was used to count spores using a hemocytometer. Afterward, spore suspension was adjusted to 10^6^ spores with 80% Tween 20 in sterile MiliQ water^44^.

Spores were germinated in GMM liquid broth supplemented with various LCOs, COs, and fatty acids at a final concentration of 10^−8^ M. All LCOs and COs stock solutions were in 0.005% aqueous ethanol. The LCOs used were as follows: sulfated C16:0 LCO, non-sulfated C16:0 LCO, sulfated C18:1 LCO, and non-sulfated C18:1 LCO. The COs used were CO4, CO5, and CO8 (IsoSep, Tullinge, Sweden). The fatty acids used were palmitic and oleic acids. The negative control for these analyses was 0.005% aqueous ethanol, the solvent in which the LCO and CO stocks were prepared. The spore concentration was adjusted to 10^6^ spores per ml of medium. One milliliter of spore suspension with the treatment of LCOs or COs were distributed into two replicate wells of a sterile Costar® 24 clear wells round, flat-bottom plate (Corning, Corning, New York) and the cells were incubated at 37 °C for 3 h. They were then observed at 1 h intervals over 21 h using a Nikon Ti inverted microscope with a ×40 objective and ten pictures were taken for each well every hour. Over 200 spores were scored for germination in each well. After 12 h of incubation, the length of the germinating apical hypha and the number of secondary branches per apical hypha were scored for over 200 germinated spores per well. Four independent experiments were performed. No differences between experiments were observed. Dose–response experiments were carried out in the same way, except that the spores were treated with a range of concentrations of sulfated C16:0 LCO(s) from 10^−6^ to 10^−13^ M.

Spores of *A. fumigatus* were grown in GMM supplemented with either 10^−8^ M sulfated C16:0 LCO or 0.005% ethanol as a negative control. The density was adjusted to 10^6^ spores per ml of medium and the cultures were maintained at 37 °C on a New Brunswick Scientific Excella E25 incubator shaker (Eppendorf, Hamburg, Germany) at 250 r.p.m. The spores were collected after 30 and 120 min by filtering the liquid broth through sterile cheesecloth. Spores were completely removed from the cheesecloth with a sterile spatula and placed into 1.5 ml Fisherbrand^TM^ Premium Microcentrifuge tubes (Thermo Fisher Scientific, Waltham, MA). Four independent cultures were replicated per treatment and time point. Immediately after spore collection, tubes were placed in liquid nitrogen for 10 min. The spores were ground to a fine powder in liquid nitrogen and transferred into 50 ml centrifuge tubes. Total RNA was extracted by using QIAzol Lysis Reagent (Qiagen, Hilden, Germany) according to the manufacturer’s instructions but with an additional phenol : chloroform : isoamyl alcohol (24 : 1 : 1) extraction step before RNA precipitation. For RNA sequencing (RNA-Seq), total RNAs were further purified by using the RNeasy Mini Kit (Qiagen). RNA samples were digested with DNase and stored at −80 °C for further use. A NanoDrop 2000 spectrophotometer (Thermo Fisher Scientific) was used to quantify and assess the purity of RNA. NanoDrop readings for samples were 112.24–491.44 ng µl^−1^.

Sixteen libraries of RNA-Seq single-end reads were prepared by using the TruSeq library preparation protocol and sequenced with an HiSeq 2500 sequencing system (Illumina, San Diego, CA). The 16 libraries corresponded to each of the four biological replicates for each of the four treatments. Read quality was assessed with FastQC 0.11.5. Read quality was excellent and adapter sequences were minimal, so reads were not trimmed. Paired-end reads were pseudo-aligned and quantified by using Kallisto 0.42.3 and the reference transcriptome of *A. fumigatus* Af293 (released 21 December 2012) downloaded from the Joint Genome Institute’s Genome Portal^53,54^. Bootstrap values were 100. Pairwise transcriptomic comparisons were made by using Sleuth version 0.30.0^55^. We defined transcripts as differentially expressed if they had a false discovery rate (*q*-value) < 0.05, *p*-value < 0.01, and *β*-values < −0.4 or >0.4. GO-enrichment analysis for the *A. fumigatus* genome was carried by using the Gene ID. GO enrichment was performed using FungiDB^56^. Potentially regulated biochemical pathways were identified using the Search Pathway feature in KEGG Mapper^57^. The search mode was set to “Afm,” to specify *A. fumigatus* as our reference organisms. DEGs for the 30 and 120 mpi timepoints were entered as search objects. A list of objects was returned (Supplementary Data 8).

### Experiments with *C. glabrata*

*C. glabrata* was grown overnight on yeast extract–peptone–dextrose medium (1% yeast extract, 2% peptone, 2% dextrose), supplemented with uridine (80 μg ml^−1^) on an orbital shaker at 200 r.p.m. and 30 °C. Ten microliters of the overnight culture were diluted 1 : 1000 in Dulbecco’s phosphate-buffered saline (without calcium or magnesium; HyClone Laboratories, Inc., Logan, UT) and counted by using a hemocytometer.

To initiate and develop biofilm production, RPMI 1640 medium (Thermo Fisher Scientific) is used for species of *Candida*^58^. Cells from an overnight culture were pelleted and resuspended (10^6^ cells per ml) in RPMI 1640 medium, supplemented with various COs, LCOs and fatty acids at a final concentration of 10^−8^ M. The LCOs, COs, fatty acids, and negative controls used were the same as for the experiments with *A. fumigatus*. Three hundred microliters of cell suspension were distributed into each well of a sterile µ-Slide 8 Well chambered coverslip (Ibidi USA, Fitchburg, WI) and the cells were observed at 10-minute intervals over 12 hours at 37 °C with 5% CO_2_ in an INU series microscope incubator (Tokhi Hit, Shizuoka-ken Japan) attached to a Nikon TI2-E inverted microscope (Nikon, Louisville KY). Each well corresponded to one treatment and five pictures were taken for each well every 10 min. After 12 h, the total number of pseudohyphae per well was counted. Four independent experiments were performed. No differences between experiments were observed. Dose–response experiments were carried out in the same way, except that the cells were treated with a range of concentrations of sulfated C16:0 LCOs from 10^−6^ to 10^−13^ M.

### Experiments with *R. mucilaginosa*

*R. mucilaginosa* strain was grown in 50 ml Difco^TM^ Dehydrated Culture Media: Potato Dextrose Broth (Thermo Fisher Scientific), in 125 ml flasks at 25 °C on an orbital shaker at 250 r.p.m. Cells were counted by using Countess^TM^ Cell Counting Chamber slides and the Countess II Automated Cell Counter (Invitrogen, Carlsbad, CA).

The concentration of *R. mucilaginosa* cells was adjusted to 10^6^ cells per ml of potato dextrose broth and various LCOs and COs were added to a final concentration of 10^−8^ M. The LCOs, COs and negative control were the same as for the experiments with *A. fumigatus* and *C. glabrata* above. Two hundred microliters of each mixture were distributed into the wells of a sterile Costar 96 Well flat-bottom plate (Corning, Corning, NY). The OD_600_ of each well was measured in 1 h intervals in a Cytation 5 Cell Imaging Multi-Mode Reader (BioTek Instruments, Winooski, VT) over 24 h at 25 °C with shaking at 0.5 r.p.m. The outer wells of the plates were filled with sterile Milli-Q water to prevent evaporation of the samples. After 24 h, the maximum V was analyzed to determine the final OD_600 nm_ reading per treatment. Six technical replicates were carried out for each treatment and three independent experiments were performed. No differences between experiments were observed.

### Prediction of proteins involved in LCO synthesis and LysM-containing proteins in fungi

The predicted number of genes encoding chitin synthases, chitin deacetylases, *N*-acyltransferases, and LysM-containing proteins was reported according to the respective references ^59–70^.

### Statistical analyses

Statistical analyses were performed using RStudio (version 1.2.1335, RStudio Team 2015, Boston, MA) and GraphPad Prism software version 8.3.0 (GraphPad, San Diego, CA). One-way analysis of variance differences were considered significant when *p* < 0.05. For the *A. fumigatus* experiments, the Tukey’s single-step multiple comparison test was used to compare the different concentrations of C16:0 sLCOs and control, and the Dunnett’s pairwise test was used to compare the treatments (LCOs and COs) to the control. Statistically significant differences were based on *p*-values < 0.05. For the *C. glabrata* experiments, the Tukey’s single-step multiple comparison test was used to compare all treatments to each other, as the control had no pseudohyphae formation, and to compare the different concentrations of C16:0 sLCO and control. For the *R. mucilaginosa* experiment, Dunnett’s pairwise test was used to compare the treatments (LCOs and COs) to the control.

### Reporting summary

Further information on research design is available in the Nature Research Reporting Summary linked to this article.

## Supplementary information

Supplementary Information

Description of Additional Supplementary Files

Supplementary Data 1

Supplementary Data 2

Supplementary Data 3

Supplementary Data 4

Supplementary Data 5

Supplementary Data 6

Supplementary Data 7

Supplementary Data 8

Supplementary Movie 1

Supplementary Movie 2

Supplementary Movie 3

Reporting Summary

## Data Availability

The RNA-seq data presented in this article are accessible through the National Center for Biotechnology Information (NCBI) BioProject# PRJNA642658 [https://www.ncbi.nlm.nih.gov/bioproject/PRJNA642658]. Source data are provided with this paper.

## Source data

Source Data
