# Supplementary material for: Lipo-chitooligosaccharides as regulatory signals of fungal growth and development
Source: Nat Commun. 2020 Aug 4;11:3897. doi: 10.1038/s41467-020-17615-5 (PMC7403392; doi:10.1038/s41467-020-17615-5)
Supplement: Supplementary file 1 — Supplementary Information [file 41467_2020_17615_MOESM1_ESM.pdf]

Supplementary Information for:

Lipo-chitooligosaccharides as regulatory signals of fungal growth and development

Rush, TA. et al.

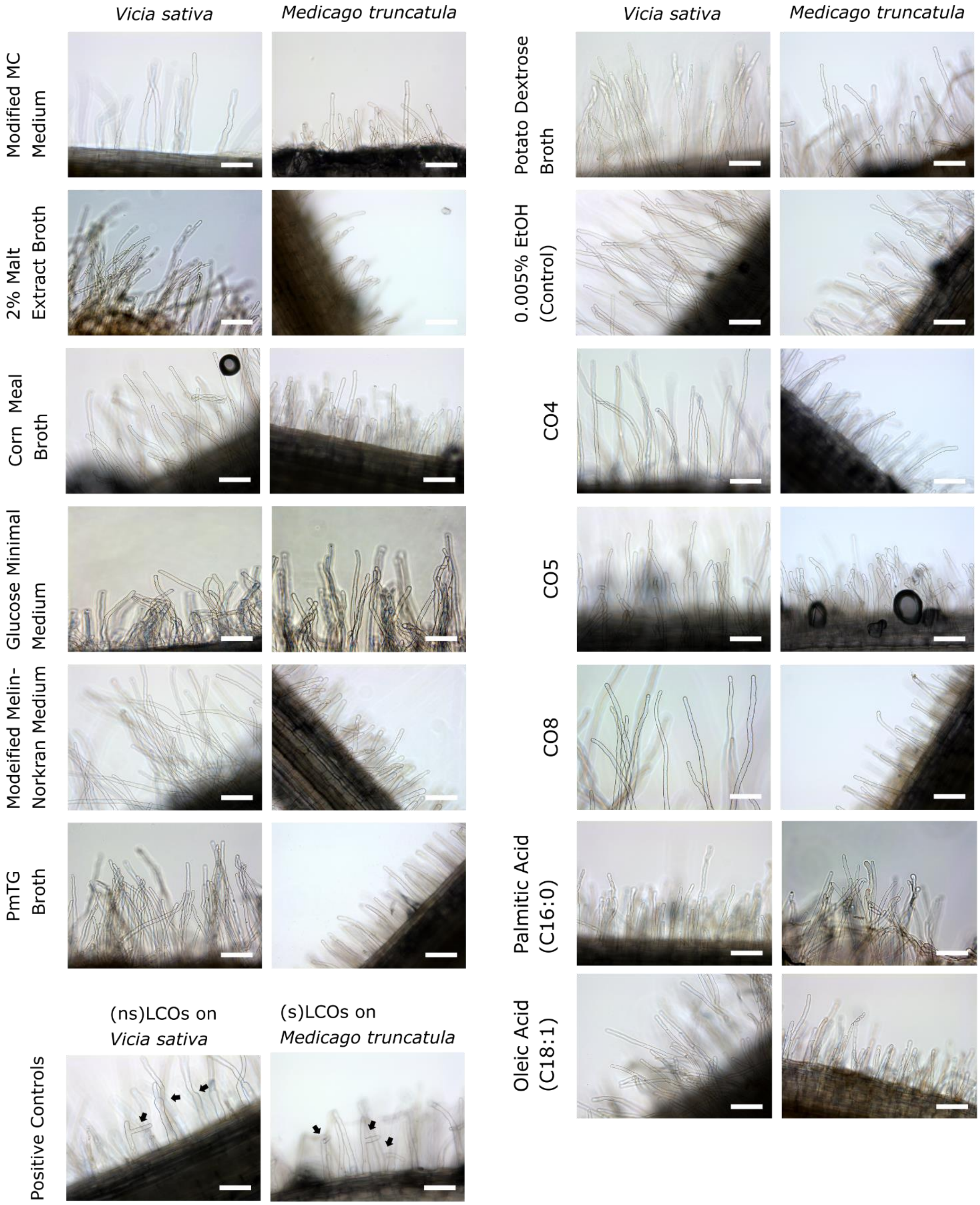
Vicia sativa
Medicago truncatula
Modified MC Medium
2% Malt Extract Broth
Corn Meal Broth
Glucose Minimal Medium
Modeified Melin-Norkran Medium
PmTG Broth
Potato Dextrose Broth
0.005% EtOH (Control)
CO4
CO5
CO8
Palmitic Acid (C16:0)
Oleic Acid (C18:1)
Positive Controls
(ns)LCOs on Vicia sativa
(s)LCOs on Medicago truncatula

Supplementary Figure 1: Root hair branching bioassay with liquid broth medium and negative control applications

Filtered liquid broth medium used for all species tested in these experiments and 0.005% of EtOH in sterile MilliQ water were applied to 1-week old seedlings of *Vicia sativa* and *Medicago truncatula* accession Jemalong A17. After 48 hours at 25ºC, roots were checked for root hair branches. Black arrows indicate that root hair branch(es) were observed, and the lack of black arrows means that no root hair branches were observed. Chitooligosaccharides and fatty acids had a concentration of 10^{-8} M. Positive controls are an application of a concentration of 10^{-8} M of (ns)LCOs on *V. sativa* and (s)LCOs on *M. truncatula*. Scale bar is 1 µm. Positive controls are an application of 10^{-8} M (ns)LCOs on *V. sativa* and (s)LCOs on *M. truncatula*.

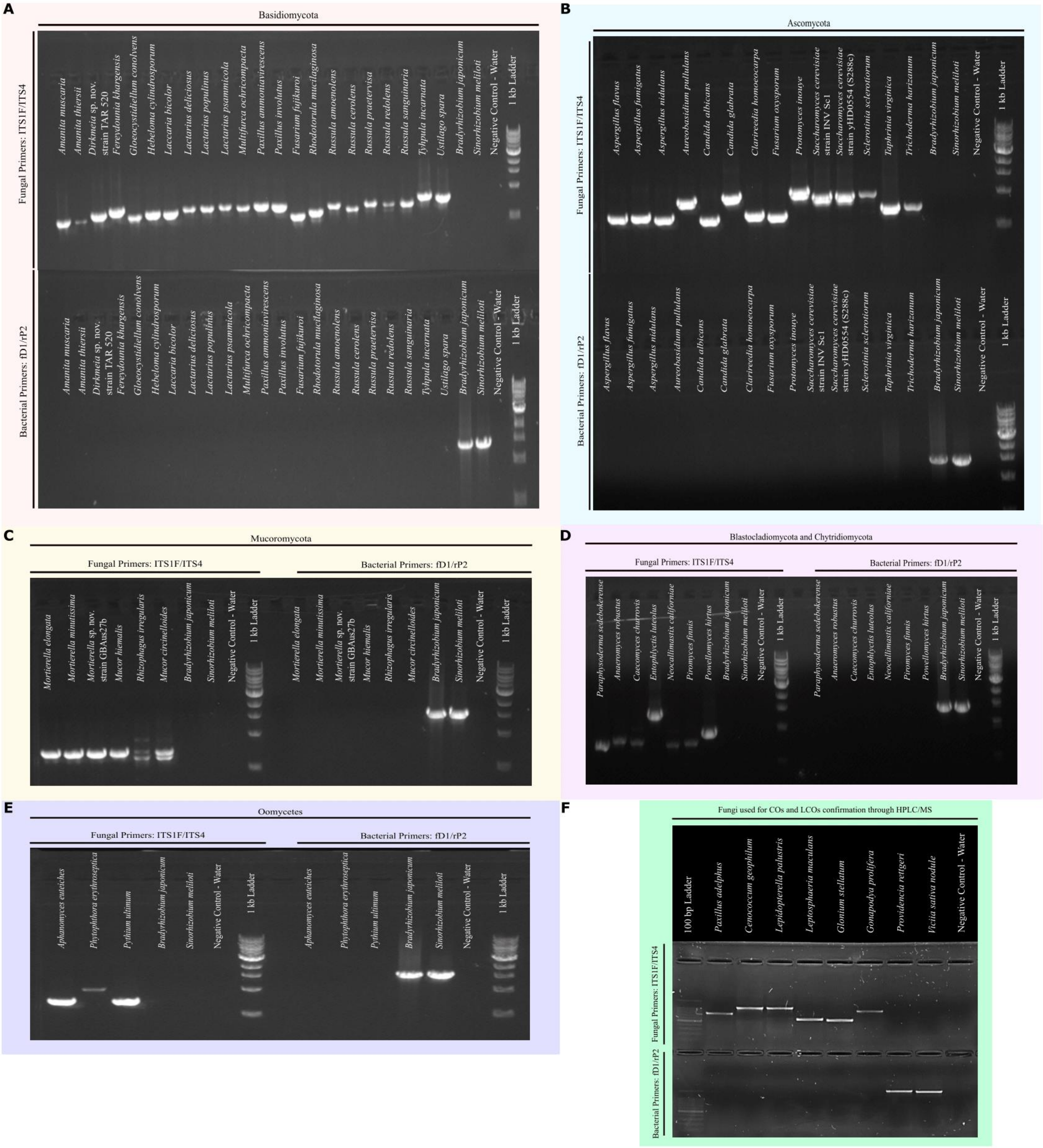
A
Basidiomycota
Fungal Primers: ITS1F/ITS4
Bacterial Primers: fD1/rP2
Amanita muscaria
Amanita thiersii
Dirkmeia sp. nov. strain TAR 520
Fereydounia khargensis
Gloeocystidiellum conolvens
Hebeloma cylindrosporum
Laccaria bicolor
Lactarius deliciosus
Lactarius populinus
Lactarius psammicola
Multifurca ochricompacta
Paxillus ammoniavirescens
Paxillus involutus
Fusarium fujikuroi
Rhodotorula mucilaginosa
Russula amoenolens
Russula cerolens
Russula praetervisa
Russula redolens
Russula sanguinaria
Tyhpula incarnata
Ustilago spara
Bradyrhizobium japonicum
Sinorhizobium meliloti
Negative Control - Water
1 kb Ladder
B
Ascomycota
Fungal Primers: ITS1F/ITS4
Bacterial Primers: fD1/rP2
Aspergillus flavus
Aspergillus fumigatus
Aspergillus nidulans
Aureobasidium pullulans
Candida albicans
Candida glabrata
Clarireedia homoeocarpa
Fusarium oxysporum
Protomyces inouye
Saccharomyces cerevisiae strain INV Sc1
Saccharomyces cerevisiae strain yHD0554 (S288c)
Sclerotinia sclerotiorum
Taphrinia virginica
Trichoderma harizanum
Bradyrhizobium japonicum
Sinorhizobium meliloti
Negative Control - Water
1 kb Ladder
C
Mucoromycota
Fungal Primers: ITS1F/ITS4
Bacterial Primers: fD1/rP2
Mortierella elongata
Mortierella minutissima
Mortierella sp. nov. strain GBAus27b
Mucor hiemalis
Rhizophagus irregularis
Mucor circinelloides
Bradyrhizobium japonicum
Sinorhizobium meliloti
Negative Control - Water
1 kb Ladder
D
Blastocladiomycota and Chytridiomycota
Fungal Primers: ITS1F/ITS4
Bacterial Primers: fD1/rP2
Paraphysoderma sedebokerense
Anaeromyces robustus
Caecomyces churrovis
Entophlyctis luteolus
Neocallimastix californiae
Piromyces finnis
Powellomyces hirtus
Bradyrhizobium japonicum
Sinorhizobium meliloti
Negative Control - Water
1 kb Ladder
E
Oomycetes
Fungal Primers: ITS1F/ITS4
Bacterial Primers: fD1/rP2
Aphanomyces euteiches
Phytophthora erythroseptica
Pythium ultimum
Bradyrhizobium japonicum
Sinorhizobium meliloti
Negative Control - Water
1 kb Ladder
F
Fungi used for COs and LCOs confirmation through HPLC/MS
Fungal Primers: ITS1F/ITS4
Bacterial Primers: fD1/rP2
100 bp Ladder
Paxillus adelphus
Cenococcum geophilum
Lepidopterella palustris
Leptosphaeria maculans
Glonium stellatum
Gonapodya prolifera
Providencia rettgeri
Vicia sativa nodule
Negative Control - Water

Supplementary Figure 2: Assessment of the absence of bacterial contamination in the culture filtrates used for root hair induction assays. DNA extracted from all samples of fungi (**A** to **D**) and oomycetes **(E)** used to detect LCOs and COs was amplified with fungi-specific ITS primers (ITS1F and ITS4)[15,16] and with bacteria-specific 16S primers (fD1/rP2)[17]. *Sinorhizobium meliloti* and *Bradyrhizobium japonicum* were used as positive controls for rhizobia. Sterile MilliQ water, which was used to make the master mix for PCR amplification, was used as a negative control. The ladder was 1 kb (New England BioLabs, Massachusetts, USA). **(F)** The absence of contamination in cultures of fungi used in the LC/MS analyses was also confirmed using fungi-specific primers (ITS1F and ITS4). Bacteria-specific primers (fD1/rP2) with *Providencia rettgeri* and *Vicia sativa* nodules inoculated with rhizobia were also used as positive controls. The ladder was benchtop 100 bp ladder (Promega).

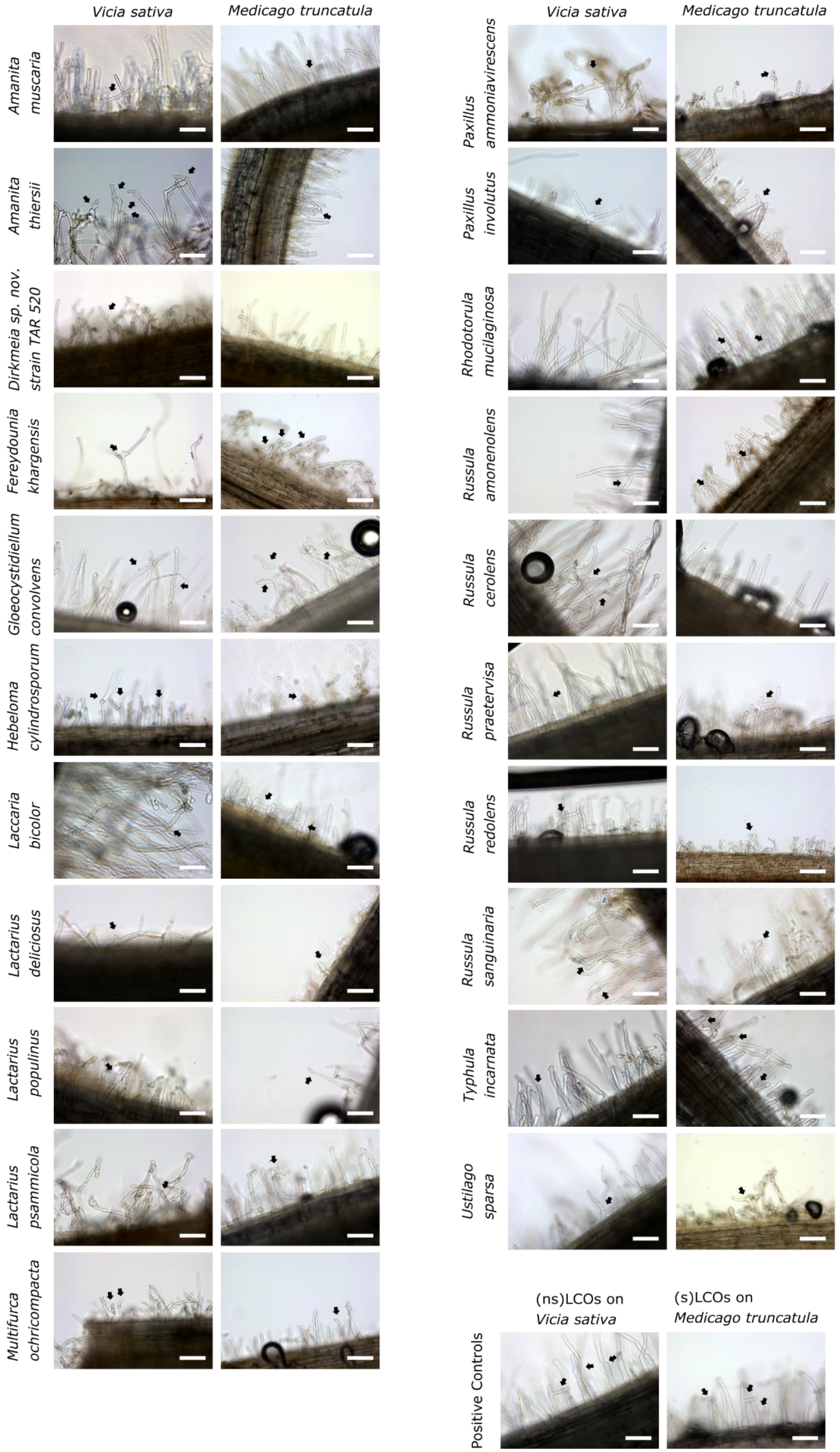

Vicia sativa
Medicago truncatula
Amanita muscaria
Amanita thiersii
Dirkmeia sp. nov. strain TAR 520
Fereydounia khargensis
Gloeocystidiellum convolvens
Hebeloma cylindrosporum
Laccaria bicolor
Lactarius deliciosus
Lactarius populinus
Lactarius psammicola
Multifurca ochricompacta
Paxillus ammoniavirescens
Paxillus involutus
Rhodotorula mucilaginosa
Russula amonenolens
Russula cerolens
Russula praetervisa
Russula redolens
Russula sanguinaria
Typhula incarnata
Ustilago sparsa
(ns)LCOs on Vicia sativa
(s)LCOs on Medicago truncatula
Positive Controls

Supplementary Figure 3: Root hair branching bioassay in Basidiomycota

Filtered exudates from basidiomycetes species were applied to 1-week old seedlings of *Vicia sativa* and *Medicago truncatula* accession Jemalong A17. After 48 hours at 25°C, roots were checked for root hair branches. Black arrows indicate that root hair branch(es), and the lack of black arrows means that no root hair branches were observed. Scale bar is 1 µm. Positive controls are an application of a concentration of 10^{-8} M of (ns)LCOs on *V. sativa* and (s)LCOs on *M. truncatula*.

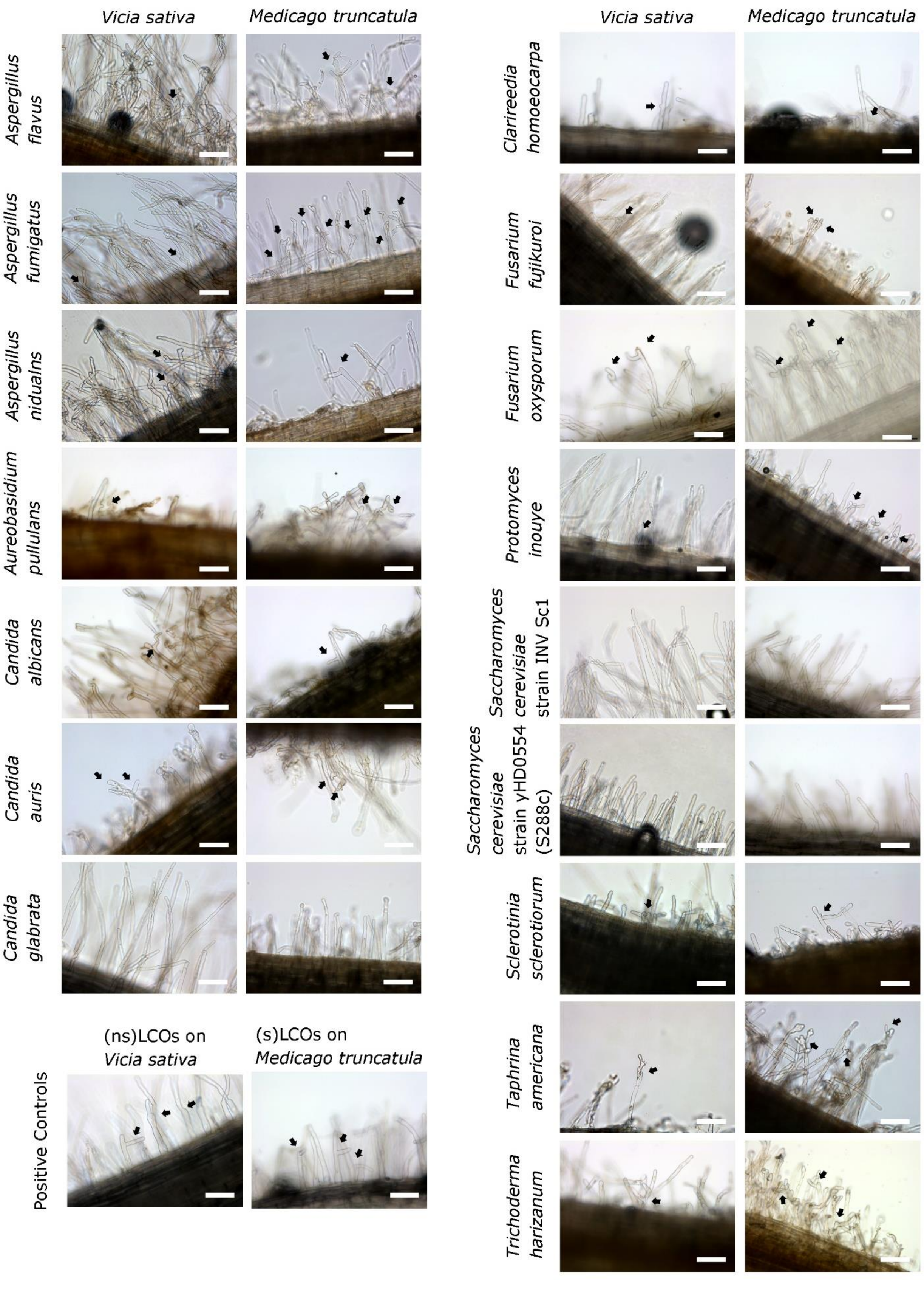

Vicia sativa
Medicago truncatula
Aspergillus flavus
Aspergillus fumigatus
Aspergillus nidualns
Aureobasidium pullulans
Candida albicans
Candida auris
Candida glabrata
Positive Controls
(ns)LCOs on Vicia sativa
(s)LCOs on Medicago truncatula
Vicia sativa
Medicago truncatula
Clarireedia homoeocarpa
Fusarium fujikuroi
Fusarium oxysporum
Protomyces inouye
Saccharomyces cerevisiae strain INV Sc1
Saccharomyces cerevisiae strain yHD0554 (S288c)
Sclerotinia sclerotiorum
Taphrina americana
Trichoderma harizanum

Supplementary Figure 4: Root hair branching bioassay in Ascomycota

Filtered exudates from ascomycete species were applied to 1-week old seedlings of *Vicia sativa* and *Medicago truncatula* accession Jemalong A17. Two different strains (yHD0554 and INV Sc1) were examined for *Saccharomyces cerevisiae.* After 48 hours at 25°C, roots were checked for root hair branches. Black arrows indicate that root hair branch(es) were observed, and the lack of black arrows means that no root hair branches were observed. Scale bar is 1 µm. Positive controls are an application of a concentration of 10^{-8} M of (ns)LCOs on *V. sativa* and (s)LCOs on *M. truncatula*.

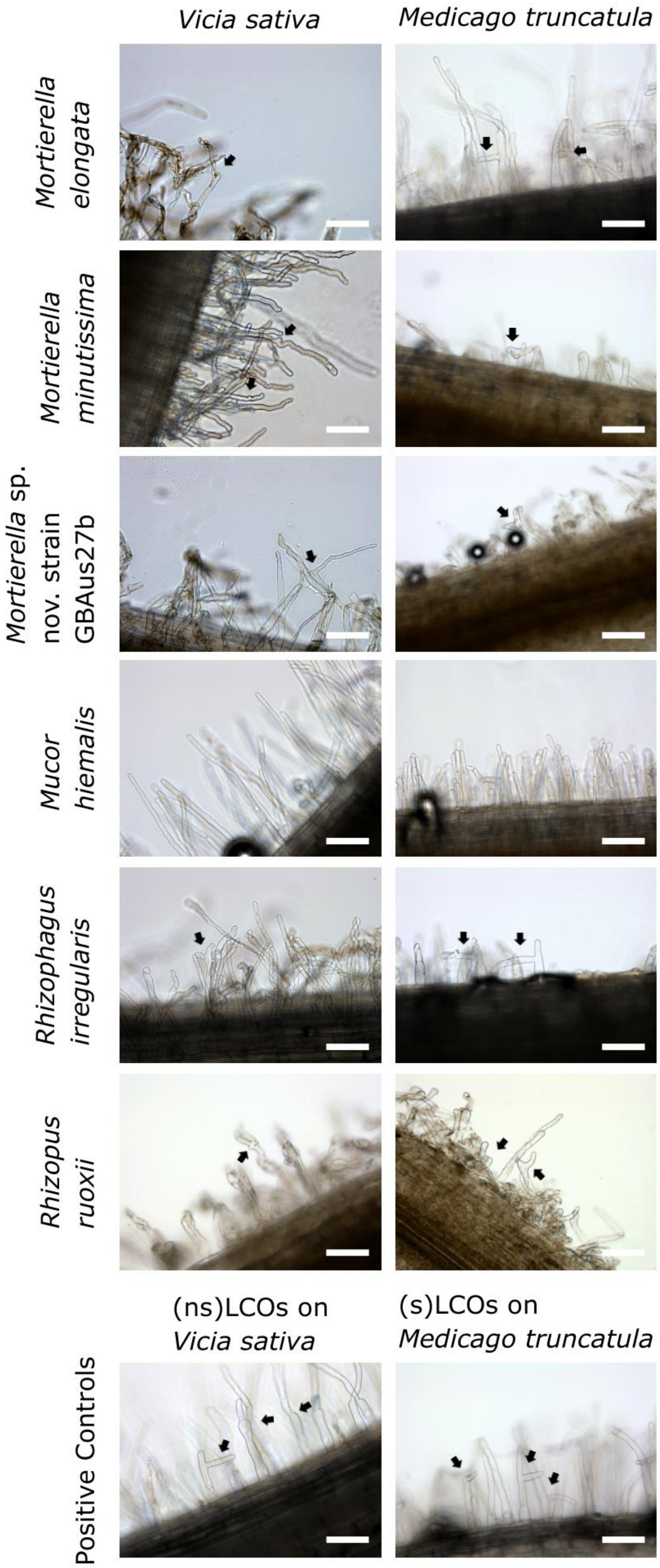

Vicia sativa
Medicago truncatula
Mortierella elongata
Mortierella minutissima
Mortierella sp. nov. strain GBAus27b
Mucor hiemalis
Rhizophagus irregularis
Rhizopus ruoxii
(ns)LCOs on Vicia sativa
(s)LCOs on Medicago truncatula
Positive Controls

Supplementary Figure 5: Root hair branching bioassay in Mucoromycota

Filtered exudates from mucoromycetes species were applied to 1-week old seedlings of *Vicia sativa* and *Medicago truncatula* accession Jemalong A17. After 48 hours at 25°C, roots were checked for root hair branches. Black arrows indicate that root hair branch(es) were observed, and the lack of black arrows means that no root hair branches were observed. Scale bar is 1 µm. Positive controls are an application of a concentration of 10^{-8} M of (ns)LCOs on *V. sativa* and (s)LCOs on *M. truncatula*.

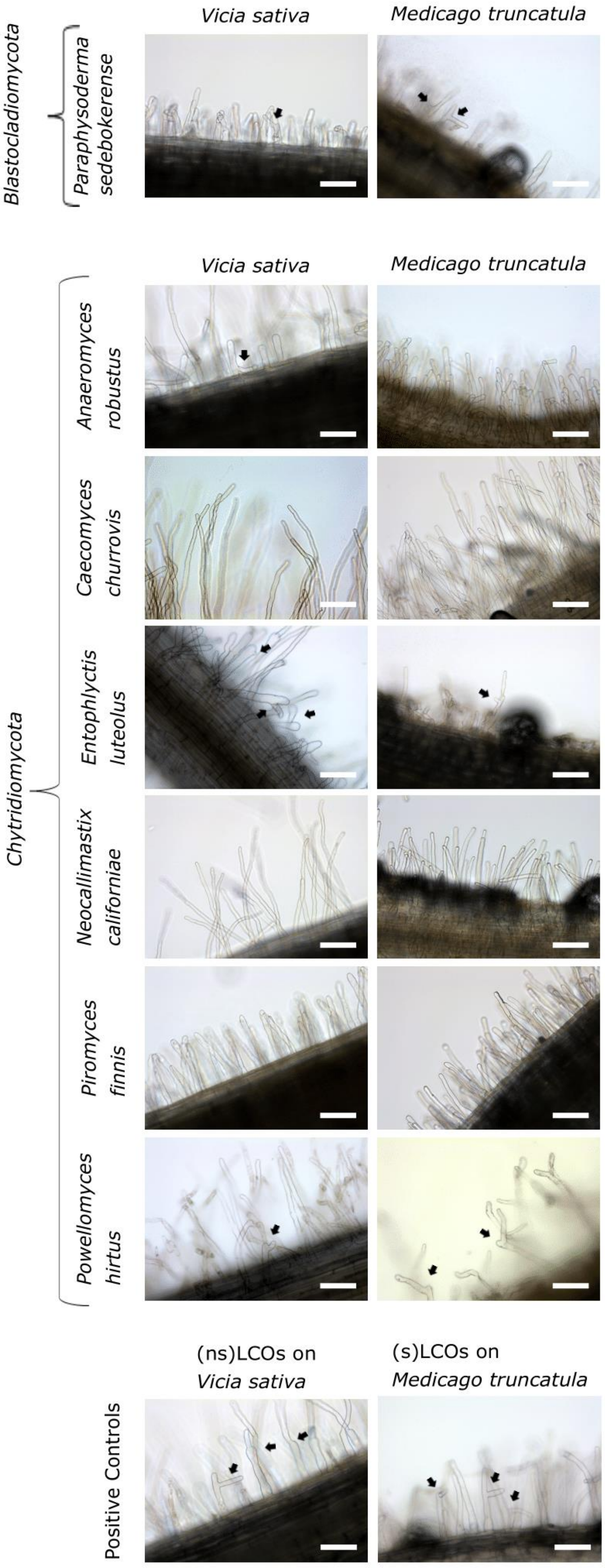

Vicia sativa
Medicago truncatula
Blastocladiomycota
Paraphysoderma sedebokerense
Chytridiomycota
Anaeromyces robustus
Caecomyces churrovis
Entophlyctis luteolus
Neocallimastix californiae
Piromyces finnis
Powellomyces hirtus
Positive Controls
(ns)LCOs on Vicia sativa
(s)LCOs on Medicago truncatula

Supplementary Figure 6: Root hair branching bioassay in Blastocladiomycota and Chytridiomycota

Filtered exudates from one species of Blastocladiomycota and six species of Chytridiomycota were applied to 1-week old seedlings of *Vicia sativa* and *Medicago truncatula* accession Jemalong A17. After 48 hours at 25°C, roots were checked for root hair branches. Black arrows indicate that root hair branch(es) were observed, and the lack of black arrows means that no root hair branches were observed. Scale bar is 1 µm. Positive controls are an application of a concentration of 10^{-8} M of (ns)LCOs on *V. sativa* and (s)LCOs on *M. truncatula*.

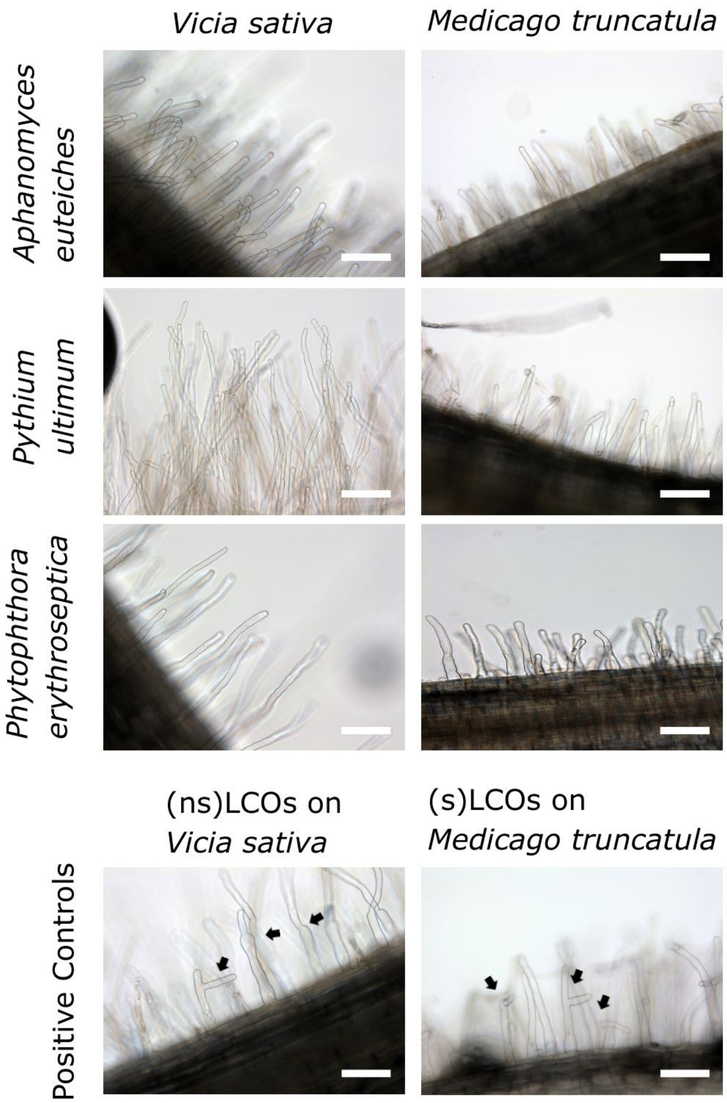

Supplementary Figure 7: Root hair branching bioassay in Oomycetes

Filtered exudates from three species were applied to 1-week old *Vicia sativa* and *Medicago truncatula* accession Jemalong A17. After 48 hours at 25°C, roots were checked for root hair branches. Black arrows indicate that root hair branch(es) were observed, and the lack of black arrows means that no root hair branches were observed. Scale bar is 1 µm. Positive controls are an application of a concentration of 10^{-8} M of (ns)LCOs on *V. sativa* and (s)LCOs on *M. truncatula*.

A

Samples/Species	Purification Level	Number of plants tested	% Responsive Plants	% Responsive plants of specific experimental negative control	% Responsive plants of specific experimental positive control	Mean intensity of response	Mean intensity of corresponding negative control	Mean intensity of corresponding positive control
Total negative controls (0.005% EtOH or 5% acetonitrile)		60	24%			0.49		
Total positive controls (*S. meliloti* Nod factors 10 nM)		73	96%			2.83		
Rhizophagus intraradices	Butanol fraction	10	90%	67%	100%	0.94	0.53	2
Gigaspora rosea	Butanol/SPE20%	8	100%	67%	100%	1.69	0.53	2
Paxillus adelphus	Butanol fraction	7	86%	57%	71%	1.43	0.325	0.89
Paxillus involutus	Butanol fraction	7	86%	57%	71%	0.17	0.325	0.89
Amanita muscaria	Butanol/Hilic 75%	10	20%	33%	100%	0.5	0.83	3.7
Cenococcum geophilum	Butanol/Hilic 75%	9	33%	33%	100%	0.5	0.83	3.7
Amanita thiersii	Butanol/Hilic 80%	7	100%	0%	100%	1.71	0	2.78
Lepidopterella palustris	Butanol/Hilic 75%	10	50%	0%	100%	1.4	0	2.78
Glonium stellatum	Butanol/Hilic 75%	10	80%	0%	100%	1.25	0	2.78

Leptopspheria maculans	Butanol/Hilic 75%	9	33%	0%	100%	1.67	0	2.78
Laccaria bicolor	Butanol fraction	8	40%	37.50%	100%	0.7	0.1	3.26
Hebeloma cylindrosporum	Butanol fraction	10	40%	37.50%	100%	0.2	0.1	3.26
Paxillus ammoniavirescens	Butanol fraction	10	60%	0%	44%	4	0	4
Gonapodya prolifea	Butanol/SPE	8	0%	0%	100%	0	0	4
Sclerotinia sclerotiorum	Butanol/SPE50%	10	30%	0%	100%	1.33	0	4
Aspergillus fumigatus	Butanol fraction	8	100%	0%	100%	0.5	0	4

B

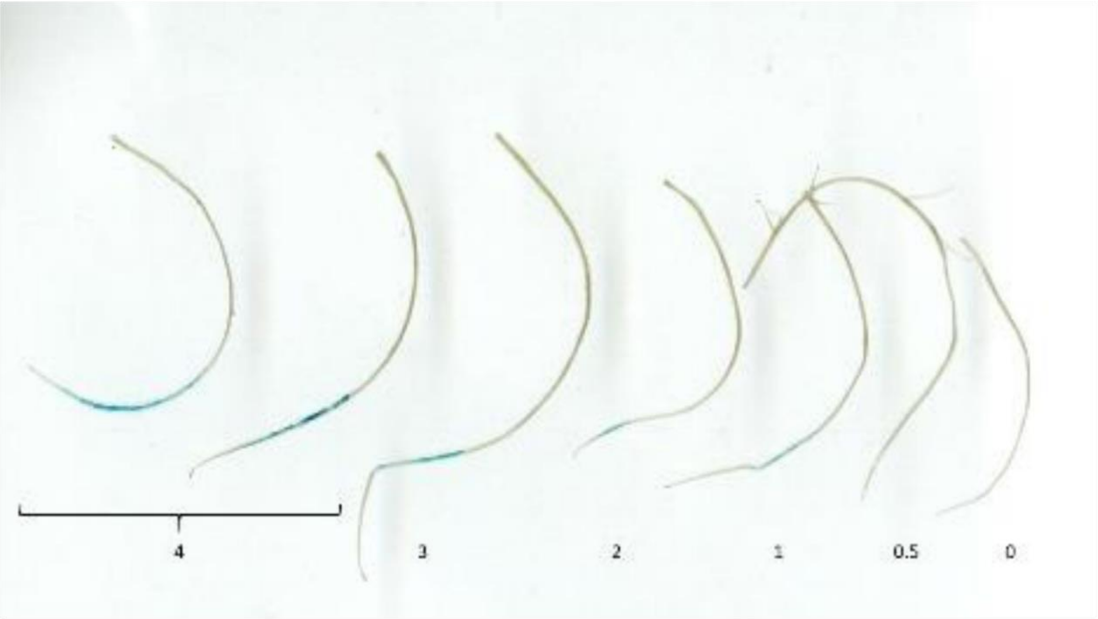

Supplementary Figure 8: *pENOD11:GUS* assay

A) The presence of sulfated LCOs in fungal exudates was assayed with the expression of *MtENOD11* in *M. truncatula* seedlings carrying a *pENOD11:GUS* transcriptional fusion. Seven to ten seedlings were used per treatment and compared to mock treatments (0.005 % EtOH in water or 5 % acetonitrile in water). Seedlings treated with Nod factors purified from *Sinorhizobium meliloti* supernatant were used as a positive control. Two kinds of samples were used, butanol extracts diluted 100 times in water and HILIC column fractions diluted 10 times. Forty microliters of the diluted fractions were applied to the primary root of each seedling for 16 hours. Staining was conducted for 6 hours. B) Arbitrary scale used to quantify the intensities of GUS staining.

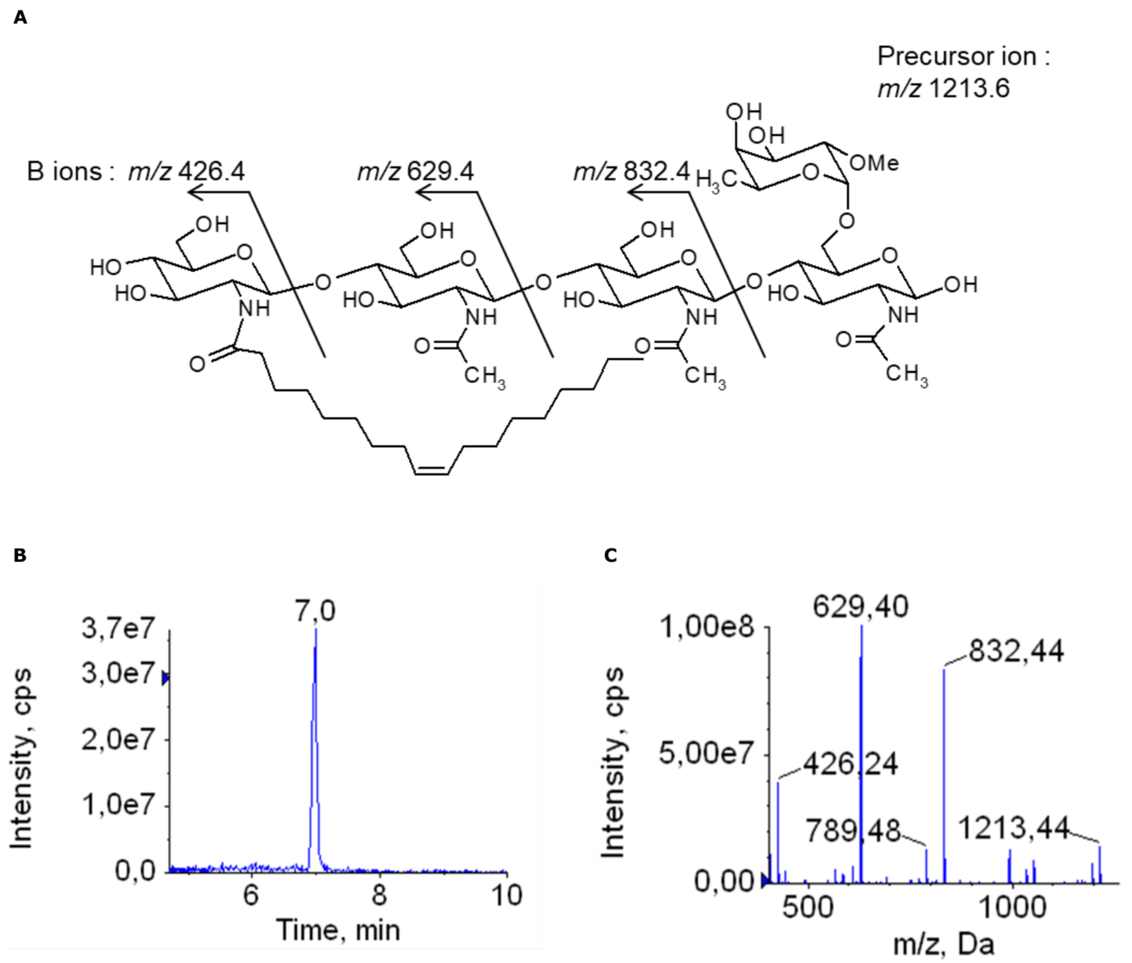

Supplementary Figure 9: Main LCO detected in butanol extract of *Gigaspora rosea* culture medium by untargeted LC-MS analysis. (**A**) Structure of the LCO IV C18:1 MeFuc. Calculated masses to charge ratio (*m/z*) of precursor and B product ions. The localization of the unsaturation on the fatty acid chain is hypothetical, as is the proposed methyl-deoxyhexose (2-methyl-fucose). (**B**) Chromatogram of the enhanced mass spectrum (EMS) of the precursor ion m/z 1213.6. (**C**) Mass spectrum at 7.0 min of the enhanced product ions (EPI) obtained from the precursor ion m/z 1213.6 using a collision energy of 40V. MeFuc: methyl-fucosyl; Da: Dalton; cps: counts per scan

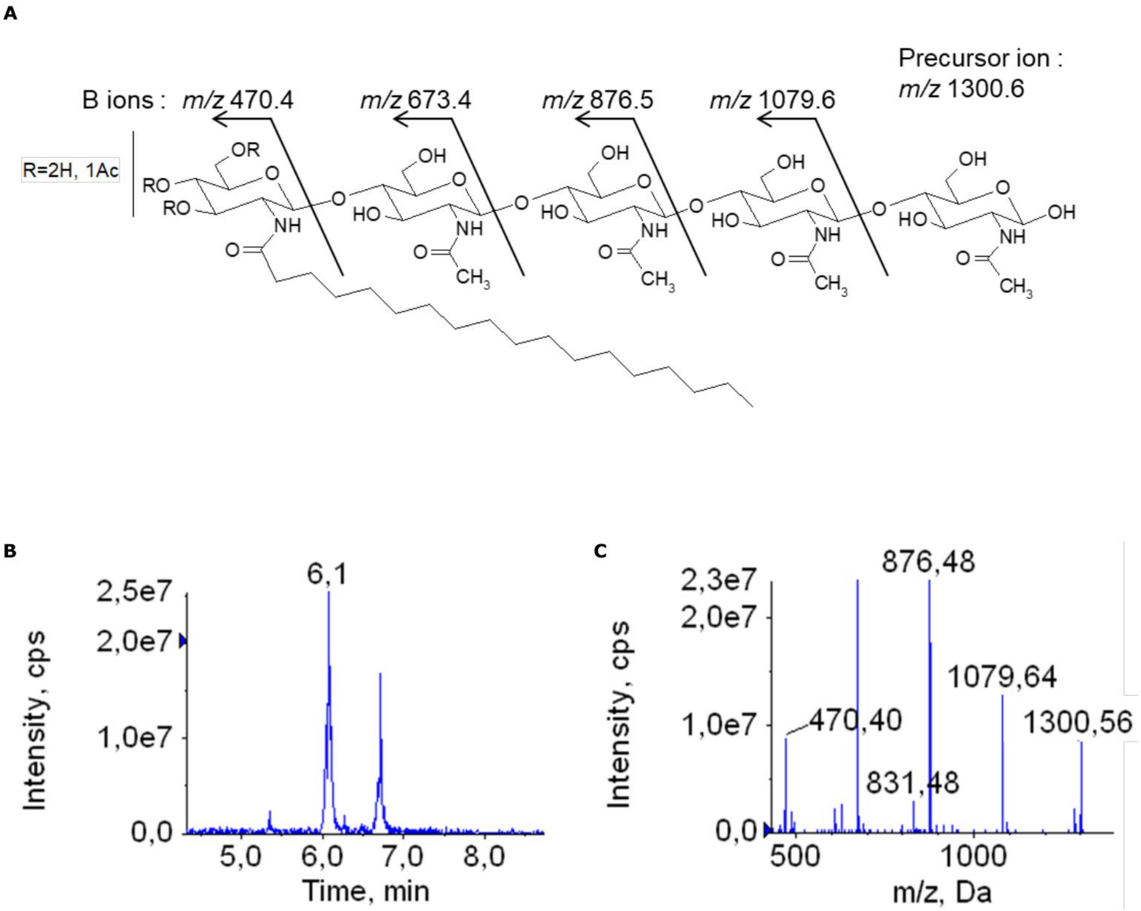

Supplementary Figure 10: Main LCO detected in butanol extract of *Paxillus adelphus* culture medium by untargeted LC-MS analysis. (**A**) Structure of the LCO V C18:0 Ac. Calculated masses to charge ratio (m/z) of precursor and B product ions. (**B**) Chromatogram of the enhanced mass spectrum (EMS) of the precursor ion m/z 1300.6. (**C**) Mass spectrum at 6.1 min of the enhanced product ions (EPI) obtained from the precursor ion m/z 1300.6 by using a collision energy of 40V. Ac: acetyl; Da: Dalton; cps: counts per scan.

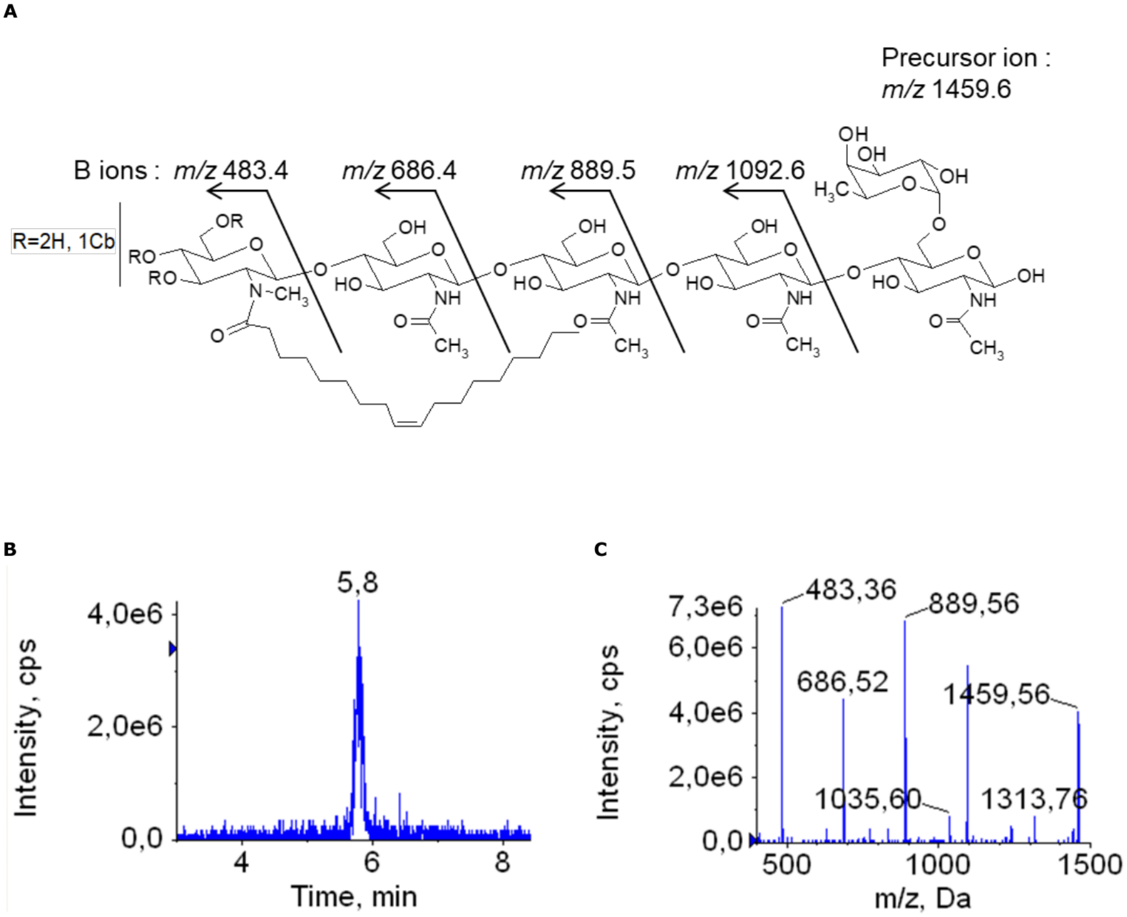

Supplementary Figure 11: Main LCO detected in butanol extract of *Paxillus involutus* culture medium by untargeted LC-MS analysis. (**A**) Structure of the LCO V C18:1 Nme, Cb, Fuc. Calculated masses to charge ratio (*m/z*) of precursor and B product ions. The location of the unsaturated double bond in the fatty acyl chain is hypothetical, as is the proposed deoxyhexose (fucose). (**B**) Chromatogram of the enhanced mass spectrum (EMS) of the precursor ion m/z 1459.6. (**C**) Mass spectrum at 5.8 min of the enhanced product ions (EPI) obtained from the precursor ion m/z 1459.6 using a collision energy of 40V. Nme: N-methyl; Cb: carbamoyl; Fuc: fucosyl; Da: Dalton; cps: counts per scan.

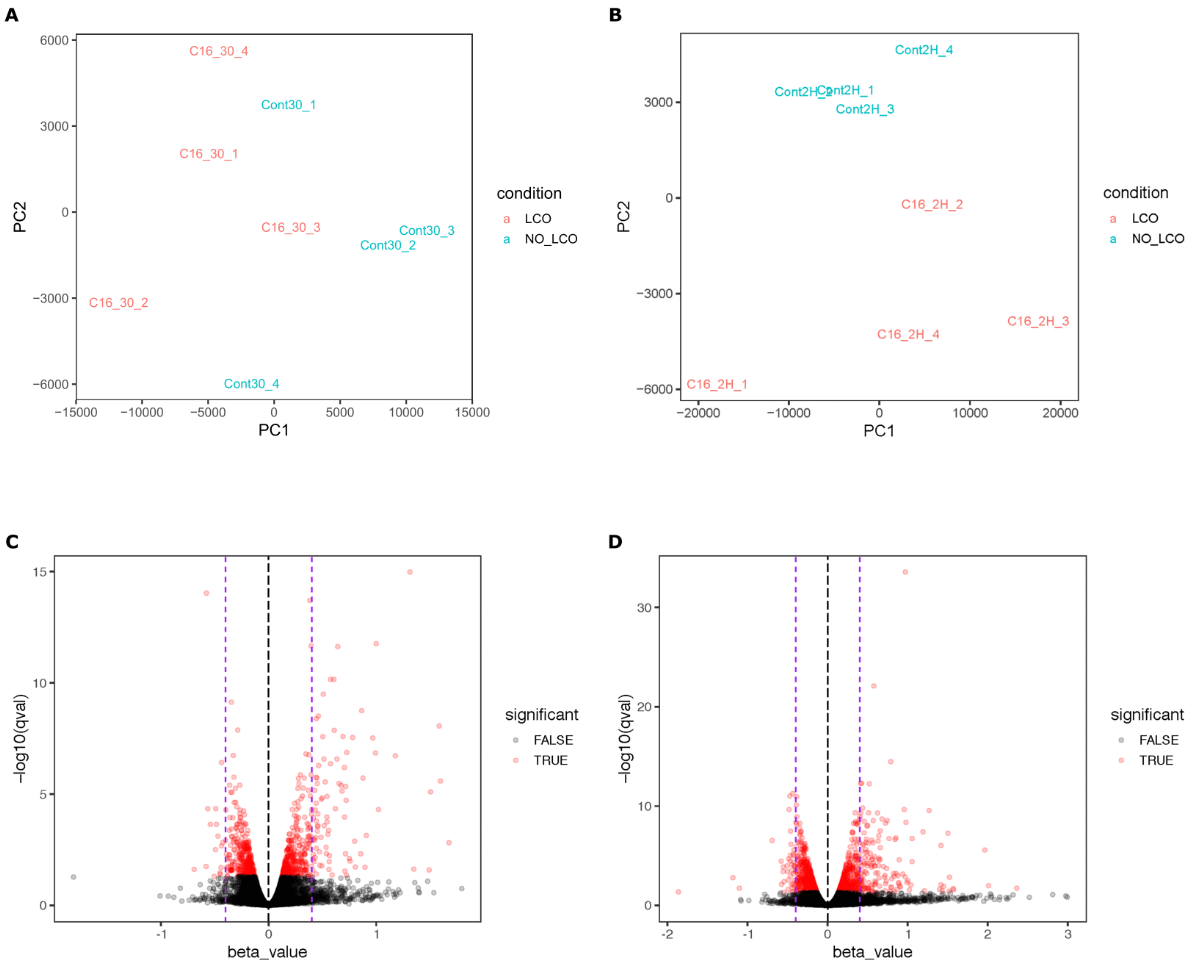

Supplementary Figure 12: Differential gene expression analysis of *Aspergillus fumigatus* treated with C16:0 sulfated LCO. Cultures of *A. fumigatus* were treated for 30 or 120 minutes with 10^{-8} M C16:0 sulfated LCO or with the solvent control (0.005% EtOH). There were four replications per treatment. An individual culture in a flask represents a single replicate. **(A)** Principal component analysis for the four replications after 30 minutes of treatment (with or without C16:0 sulfated LCOs) showing that replicates are similar and that the LCO-treatment had a significant effect (**B**) Significant DEGs with the -log10(q-value) and beta value for genes expressed below -0.4 or higher than 0.4 after 30 minutes. **(C)** Principal component analysis for the four replications (cultures in different flasks) at 120 minutes post-treatment (with or without C16:0 sulfated LCOs) showing that replicates are similar and that the LCO-treatment had a significant effect **(D**) Significant DEGs with the -log10(q-value) and beta value for genes expressed below -0.4 or higher than 0.4 after 120 minutes.

a

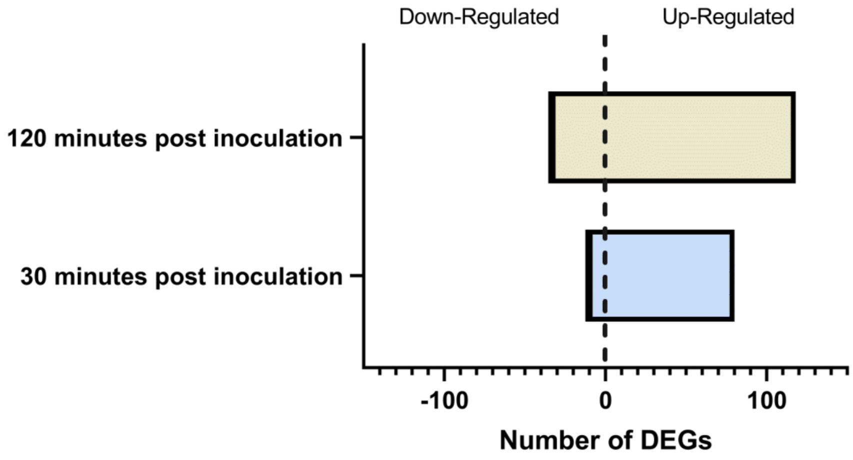
Down-Regulated
Up-Regulated
120 minutes post inoculation
30 minutes post inoculation
-100
0
100
Number of DEGs

b

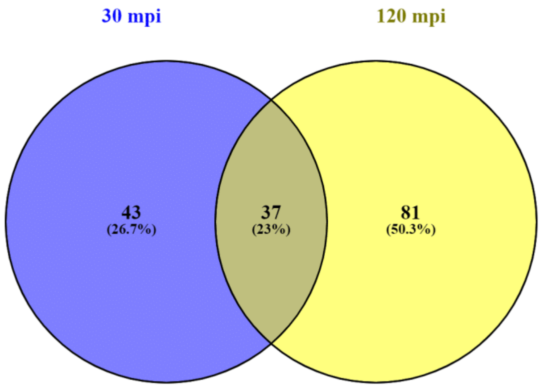
30 mpi
120 mpi
43
(26.7%)
37
(23%)
81
(50.3%)

c

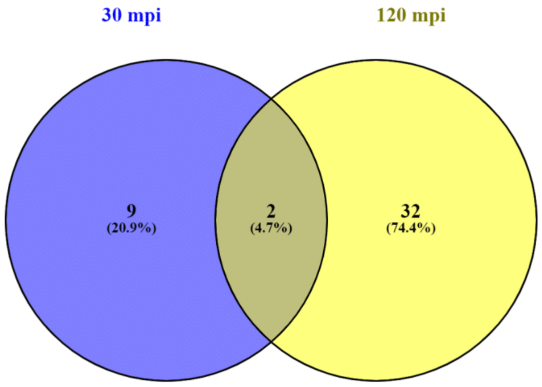
30 mpi
120 mpi
9
(20.9%)
2
(4.7%)
32
(74.4%)

Supplementary Figure 13: Shared DEGs produced after 30- and 120- mpi

(a) Regulation of DEGs from 30- and 120- mpi. 80 genes were up-regulated, and 11 genes were down-regulated after 30mpi. 118 genes were up-regulated, and 34 genes were down-regulated after 120mpi. (b) Up-regulated DEGs found in 30mpi (blue), 120mpi (yellow) and shared. The shared genes are:Afu5g02500, Afu6g10940, Afu6g06590, Afu1g04310, Afu6g08270, Afu1g04300, Afu2g17320, Afu6g08940, Afu1g01350, Afu6g14270, Afu4g09330, Afu2g08280, Afu5g08650, Afu7g06770, Afu3g14260, Afu8g07225, Afu3g03940, Afu5g12410, Afu1g02040, Afu7g02050, Afu2g09070, Afu1g00500, Afu1g06740, Afu6g00500, Afu4g03460, Afu6g06640, Afu4g11720, Afu1g04270, Afu3g14230, Afu3g03280, Afu1g09580, Afu2g02500, Afu6g07910, Afu8g00680, Afu6g07720, Afu2g05060, Afu1g09300. (c) Down-regulated DEGs found in 30mpi (blue), 120mpi (yellow) and shared. The shared genes are Afu5g02800 and Afu1g02590.

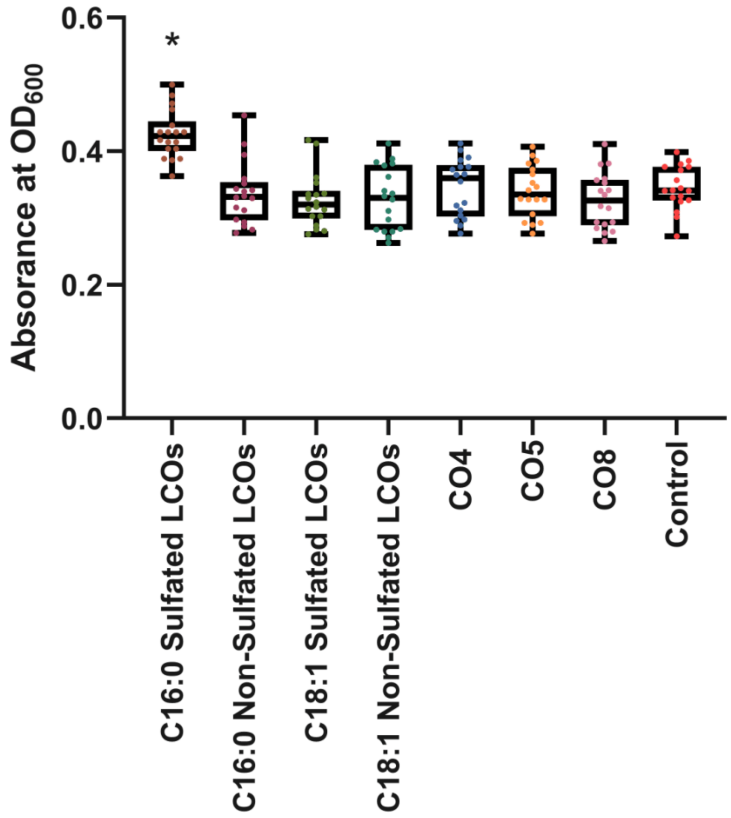

Supplementary Figure 14: Effect of LCOs and COs on *Rhodotorula mucilaginosa* cell growth. Growth determined by OD_{600} reading after 24-hour treatment with 10^{-8} M LCOs and COs. The data are from three experiments, each with six samples per treatment. The liquid broth medium was potato dextrose. (*) indicates a significant difference between C16:0 sulfated LCOs and the control; one-way ANOVA p-value was 2.96 $x10^{-11}$. Dunnett's multiple comparison procedures was used. 18 wells were analyzed per treatment. In the box plot, the bars represent the minimum value, the first quartile, the third quartile, and the maximum value such that 25% of the data are in each section. Source data are provided as a Source Data file.

Fungi	Proteins related to chitin synthesis and binding			
**Ascomycetes**	**Chitin synthases**	**Chitin deactylases**	**N-acyltransferases***	**LysM proteins***
Aspergillus fumigatus	8[59,62]	7[63]	73[69]	9[68]
Botrytis cinerea	8[59]/6[62]	9	73[69]	6[68]
Pochonia chlamydosporia	8[64]	3[64]	131[69]	8[69]
Trichoderma atroviride	8[68]	6[69]	93[69]	12[68]
**Basidiomycetes**				
Ustilago maydis	8[59]/6[60, 62]	6[67]	56[69]	2[68]
Moniliophthora roreri	9[60]	13[67]	11[70]	1[70]
Coprinopsis cinerea	9[59,60,62]	17[67]	54[69]	5[68]
Laccaria bicolor	10[59]/8[60,62]	10	57[69]	4[68]
**Zygomycetes**				
Rhizopus stolonifer	57[65]	28[65]/29[66]	65[69]	12[69]
Rhizopus delemar	26[59]/19[60]/23[61]/20[62]	30-37[66]	64[69]	11[69]
Batrachochytrium dendrobatidis	15[59,60,62]	9[67]	29[69]	2[68]

Supplementary Table 1: Predicted number of genes encoding chitin synthases, chitin deacetylases, N-acyltransferases and LysM containing proteins in selected fungi

The number of proteins shown is reported in the respective references[49-60]. (*) The number of N-acyltransferases and LysM proteins is based on annotation in JGI's Mycocosm portal, except for *Moniliophthora roreri*, for which the numbers are based on annotation in NCBI.
