# Supplementary material for: Lipo-chitooligosaccharides as regulatory signals of fungal growth and development
Source: Nat Commun. 2020 Aug 4;11:3897. doi: 10.1038/s41467-020-17615-5 (PMC7403392; doi:10.1038/s41467-020-17615-5)
Supplement: Supplementary file 2 — Description of Additional Supplementary Files [file 41467_2020_17615_MOESM2_ESM.pdf]

DESCRIPTION OF ADDITIONAL SUPPLEMENTARY FILES

Supplementary Data 1: Origin of fungal and oomycete cultures

Supplementary Data 2: Culture conditions and media used

Supplementary Data 3: Taxonomic affiliation and lifestyle

Supplementary Data 4: Number of root hair branches per treatment and controls. The total number of root hair branches observed in 3-cm of root were recorded for a minimum of five replications per fungal and oomycete species and controls tested on *Medicago truncatula* and *Vicia sativa*. Chitooligosaccharides and fatty acids had a concentration of 10^{-8} M. Positive controls are an application of a concentration of 10^{-8} M of (ns)LCOs on *V. sativa* and (s)LCOs on *M. truncatula*. Broth media were also examined.

Supplementary Data 5: Database listing all possible mass-to-charge ratios (precursors/products ions) calculated from known LCOs.

Supplementary Data 6: List of Gene Ontology (GO) Enrichment Terms and Differentially Expressed Genes for 30- and 120-minutes post inoculation. The GO Enrichment terms were separated into three functions: biological process, cellular component, and molecular function. Significant DEGs with genes expressed with a p-value <0.05 after 30 minutes or after 120 minutes.

Supplementary Data 7: GO Enrichment Terms 30- and 120-minutes post inoculation:

Supplementary Data 8: DEGs in KEGG pathway after 120 minutes post inoculation

Supplementary Movie 1: Formation of pseudohyphae and swollen cells in *Candida glabrata*
Swollen cells and pseudohyphae observed 10 hours after treatment with 10^{-8} M C18:1 sulfated LCOs. Time-lapse bright field microscopy was used. Scale bar is 50 μm.

Supplementary Movie 2: A closer view of pseudohyphae formation in *Candida glabrata*
Pseudohyphae were observed 10 hours after treatment with 10^{-9} M C16:0 sulfated LCOs. Time-lapse bright field microscopy was used. Scale bar is 10 μm.

Supplementary Movie 3: Z-Stack of extensive pseudohyphae formation in *Candida glabrata*
3D image constructed from a z stack showing extensive pseudohyphae formation 10 hours after treatment with 10^{-9} M C16:0 sulfated LCOs. Time-lapse bright field microscopy was used. The X-axis shows increments of 50 μm (up to a total of 233 μm) as the scale bar at 40X magnification.
